# The membrane-active polyaminoisoprenyl compound NV716 re-sensitizes *Pseudomonas aeruginosa* to antibiotics and reduces bacterial virulence

**DOI:** 10.1038/s42003-022-03836-5

**Published:** 2022-08-25

**Authors:** Gang Wang, Jean-Michel Brunel, Matthias Preusse, Negar Mozaheb, Sven D. Willger, Gerald Larrouy-Maumus, Pieter Baatsen, Susanne Häussler, Jean-Michel Bolla, Françoise Van Bambeke

**Affiliations:** 1 Pharmacologie cellulaire et moléculaire, Louvain Drug Research Institute, Université catholique de Louvain, Brussels, Belgium; 2 Aix Marseille Université, INSERM, SSA, Membranes et Cibles thérapeutiques (MCT), Marseille, France; 3 Department of Molecular Bacteriology, Helmoltz Centre for Infection Research, Braunschweig, Germany; 4 Department of Molecular Bacteriology, Twincore, Hannover, Germany; 5 Department of Life Sciences, Faculty of Natural Sciences, MRC Centre for Molecular Bacteriology and Infection, Imperial College London, London, United Kingdom; 6 Electron Microscopy Platform & Bio Imaging Core, VIB & KULeuven Center for Brain & Disease Research, KULeuven, Leuven, Belgium; 7 Department of Clinical Microbiology, Rigshospitalet, Copenhagen, Denmark; 8 Cluster of Excellence RESIST, Hannover Medical School, Hannover, Germany; 9 Present Address: Institute for Medical Biometry and Bioinformatics, Medical Faculty, Heinrich Heine University Düsseldorf, Düsseldorf, Germany

**Keywords:** Pharmacodynamics, Antibiotics

## Abstract

*Pseudomonas aeruginosa* is intrinsically resistant to many antibiotics due to the impermeability of its outer membrane and to the constitutive expression of efflux pumps. Here, we show that the polyaminoisoprenyl compound NV716 at sub-MIC concentrations re-sensitizes *P. aeruginosa* to abandoned antibiotics by binding to the lipopolysaccharides (LPS) of the outer membrane, permeabilizing this membrane and increasing antibiotic accumulation inside the bacteria. It also prevents selection of resistance to antibiotics and increases their activity against biofilms. No stable resistance could be selected to NV716-itself after serial passages with subinhibitory concentrations, but the transcriptome of the resulting daughter cells shows an upregulation of genes involved in the synthesis of lipid A and LPS, and a downregulation of quorum sensing-related genes. Accordingly, NV716 also reduces motility, virulence factors production, and biofilm formation. NV716 shows a unique and highly promising profile of activity when used alone or in combination with antibiotics against *P. aeruginosa*, combining in a single molecule anti-virulence and potentiator effects. Additional work is required to more thoroughly understand the various functions of NV716.

## Abstract (web summary)

The polyaminoisoprenyl compound NV716 re-sensitizes *Pseudomonas aeruginosa* to antibiotics through permeabilizing the outer membrane and increases the activity of antibiotics on biofilms.

## Introduction

The Gram-negative bacterium *Pseudomonas aeruginosa* is an important opportunistic pathogen that may cause life-threatening infections. These are difficult to treat because *P.aeruginosa* has developed many mechanisms of resistance to currently-used antibiotics. Beside acquired resistance mechanisms, like the production of enzymes modifying or hydrolyzing antibiotics and mutations in antibiotic targets, intrinsic resistance mediated by the poor permeability of the outer membrane to many antibiotics as well as by the expression of multidrug efflux pumps^1^ contributes to restrict the number of antibiotic classes showing useful activity against this species. This is notably the case for a series of old, abandoned drugs, otherwise characterized by a broad spectrum of activity.

The envelope of *P.aeruginosa* consists of three layers: the inner membrane (IM), made of a fluid phospholipid bilayer, the periplasmic space containing the peptidoglycan cell wall, and the outer membrane (OM), a hallmark of Gram-negative bacteria. The outer membrane is structurally asymmetric, with its inner leaflet containing phospholipids, and the outer leaflet, made of lipopolysaccharides (LPS). LPS molecules comprise a polysaccharidic antigenic part, a core oligosaccharide and a lipidic part (lipid A) insuring the anchoring in the membrane. LPS packing is enhanced by the intercalation of divalent cations such as Mg^2+^ and Ca^2+^ that neutralize negative charges within the LPS molecules^2^. This assemblage contributes to the structural integrity of the bacteria, and opposes a barrier to the diffusion of lipophilic or of high-molecular-weight compounds^2^.

In addition, this envelope contains a series of transmembrane proteins, among which different efflux systems belonging to the resistance-nodulation-division (RND) superfamily. These efflux systems are made of three proteins spanning the entire envelope, including an efflux transporter embedded in the inner membrane, a periplasmic membrane fusion protein, and an outer membrane channel. Among these transporters, MexAB-OprM, MexCD-OprJ, MexEF-OprN, and MexXY-OprM are those mainly contributing to multidrug resistance by extruding different structurally-unrelated classes of antibiotics^3^. Almost all antibiotics are substrates for active efflux in *P. aeruginosa*^3^, increasing the MIC of many of them above clinically-achievable levels, although these drugs show high affinity for their target in acellular systems, as demonstrated for example for tetracyclines or chloramphenicol^4,5^.

Permeabilizing the outer membrane while at the same time impairing active efflux, therefore appears as an appealing strategy to improve the susceptibility to antibiotics of Gram-negative bacteria, including *P. aeruginosa*^6^. In this respect, a promising approach consists in combining antibiotics with potentiators interfering with these barrier effects.

The efflux pump inhibitor Phenyl-Arginine-β-naphthylamide (PAβN) and the outer membrane permeabilizer polymyxin nonapeptide (PMBN) have been widely used in-vitro to increase the potency of antibiotics towards Gram-negative bacteria^7,8^, but their toxicity and inadequate pharmacokinetic properties hinder clinical applications^9,10^. Efforts have thus been made to synthesize new adjuvant molecules with improved safety profile and enhanced potency^11^. Among them, the polyaminoisoprenyl compounds NV716 and NV731 proved capable to restore the activity of chloramphenicol towards *Enterobacteria spp*^12^ and one of them (NV716), that of chloramphenicol and doxycycline towards *P. aeruginosa*^13^ or florfenicol towards *Bordetella bronchiseptica*^14^. Moreover, NV716 also improved the potency and the efficacy of doxycycline, chloramphenicol, rifampicin, and ciprofloxacin against intracellular forms of *P. aeruginosa* by its capacity to inhibit efflux and to reduce the fraction of persisters in the bacterial population^15^. Previous mechanistic studies suggest a complex mode of action, including the inhibition of the activity of efflux pumps and an alteration of the outer membrane permeability barrier^13^ at concentrations that do not alter eukaryotic cell viability^15^.

The aim of the present study was to examine in details the effects of NV716 (see chemical structure in Supplementary Fig. 1) on the activity of abandoned antibiotics against *P. aeruginosa*, namely doxycycline, chloramphenicol (both substrates for efflux^3^), rifampicin (poor substrate for efflux^16^ but showing low outer membrane permeation^17^), and of ciprofloxacin, selected as an active drug against *P. aeruginosa*. We also characterized in details its interaction with the bacterial envelope, its capacity to prevent selection of resistance, and to improve antibiotic activity against biofilms. Lastly, we performed a genomic and transcriptomic analysis of bacteria exposed for serial passages to this molecule in order to get more insight on its mechanism of action. Throughout this work, we compared NV716 with NV731 (less potent against *P. aeruginosa*^13^) and PAβN (as a reference efflux pump inhibitor). We also included in our study colistin, PMBN, and alexidine as positive controls, as they all show well-characterized effects on *P.aeruginosa* envelope (see Supplementary Fig. 1 for the structure of all these compounds). The polymyxin colistin binds to the outer membrane via electrostatic interactions with the negatively-charged LPS molecules, more specifically with the lipid A component. By displacing Mg^2+^ and Ca^2+^, polymyxins destabilize the outer membrane, cross it via a self-promoted uptake mechanism, and subsequently insert in the inner membrane and disrupt the physical integrity of the phospholipid bilayer^18^. PMBN nonapeptide (i.e., a derivative of polymyxin B lacking the lipophilic tail) also increases outer membrane permeability by binding with high affinity to LPS and lipid A via electrostatic interactions. Nevertheless it cannot establish hydrophobic interactions with lipid A, explaining why it is a poor antibacterial compound, but it is still synergistic with hydrophobic antibiotics by increasing their capacity to cross the outer membrane barrier^19^. Alexidine is a biguanide antiseptic showing a rapid bactericidal effect^20^. As polymyxins, it establishes electrostatic interactions with the negative charges of LPS, displacing Mg^2+^ from its binding to LPS and disrupting the stabilizing effect afforded by Mg^2+^ cross-bridging of adjacent LPS molecules^21^. In addition, it also causes the leakage of the cytoplasmic content by interacting with membrane lipids, inducing the formation of lipid domains^22^.

We show that NV716, contrarily to its comparators, markedly decreased the MICs of all antibiotics against *P. aeruginosa*, whether substrates or not for efflux, and in strains expressing or not efflux pumps. These effects could be attributed to a strong interaction with LPS and disturbance of outer membrane integrity that increased the intracellular accumulation of antibiotics. Beside this potentiator effect, NV716 prevents the selection of resistance, improves antibiotic activity against biofilms, prevents biofilm formation, reduces motility and production of virulence factors. Thus, NV716 shows a unique and promising profile of activity, combining in a single molecule the beneficial effects of membrane-disturbing agents (like alexidine and PMBN) and efflux pumps inhibitors (like PAβN) and demonstrating at the same time additional useful adjuvant properties, while proving untoxic to eukaryotic cells and avoiding the risk of resistance selection observed with colistin.

## Results

### Antimicrobial susceptibility

We first examined to capacity of NV716 to potentiate antibiotic activity. Supplementary Data 1 shows the MIC of antibiotics alone or combined with potentiators against 4 reference strains and 71 clinical isolates, among which, 4 resistant to colistin.

**Fig. 1:**
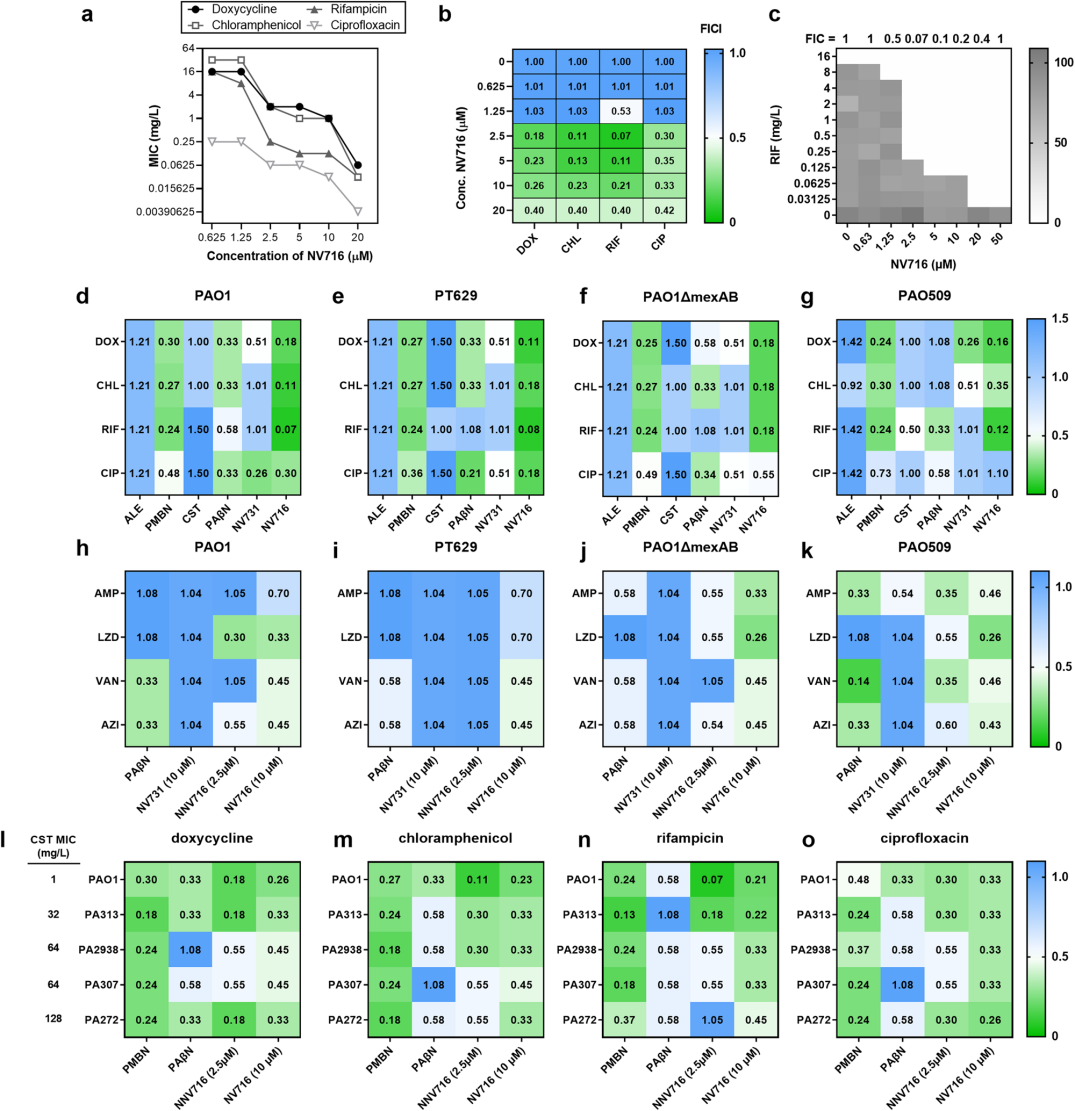
Checkerboard analyses for the evaluation of the synergy between potentiators and antibiotics. **a**–**c** Detailed analysis for PAO1. **a** Change in the MICs of 4 antibiotics in the presence of NV716 at increasing concentrations. **b** Determination of the corresponding FIC indexes (FICI) in a heatmap plot. **c** Checkerboard plate having allowed to determine FICI for the combination of NV716 and rifampicin. The gray scale shows the A_620 nm_ in percentage of the maximal value. The other panels show heatmaps describing FICI of potentiators for reference strains in combination with (**d**–**g**) abandoned antibiotics (DOX doxycycline, CHL chloramphenicol, RIF rifampicin) and ciprofloxacin (CIP), calculated for concentrations of 2.5 μM alexidine, 0.5 µM colistin (CST), 30 µM PMBN, 38 µM PAβN, 2.5 μM NV731 and 2.5 μM NV716; or with (**h**–**k**) ampicillin (AMP) or antibiotics active against Gram-positive bacteria (LZD linezolid, VAN vancomycin, AZI azithromycin), calculated for concentrations of 38 µM PAβN, 2.5 μM NV731, 2.5 mM and 10 μM NV716; or for 4 colistin-resistant isolates^25^ *vs*. PAO1 (**l**–**o**, with colistin MICs are shown on the left) combined with the same antibiotics as in **d**–**g** and calculated for concentrations of 38 µM PAβN, 30 µM PMBN, or 2.5 or 10 µM NV716. Synergy is defined as FICI < 0.5 (appearing in green on the graphs). All data are mean from at least two independent experiments. PT629: MexAB overproducer derivative of PAO1; PAO1*mexAB*: PAO1 derivative deleted for the expression of *mexAB;* PAO509: PAO1 derivative deleted for the expression of 5 RND efflux transporters; PA313, PA2938, PA307, PA272: colistin-resistant isolates.

In reference strains, the MIC of chloramphenicol, doxycycline and ciprofloxacin against PAO1 and PT629 (MexAB-OprM overproducer) was higher than against PAO1*mexAB* (∆*mexAB*) and PAO509 (deleted in the genes encoding 5 efflux pumps), indicating that these three antibiotics are substrates for efflux. The MIC of rifampicin alone was the same in the four reference strains, confirming that this drug is a poor substrate for efflux pumps. Alexidine (2.5 µM) and colistin (0.5 µM; subMIC concentration) did not affect the MIC of all tested antibiotics. PAβN (38 μM) and NV731 (2.5 and 10 μM) reduced antibiotic MICs up to 4-fold, the lowest effect being observed in combination with rifampicin. Polymyxin B nonapeptide (PMBN; 30 μM) and NV716 (2.5 and 10 μM) systematically decreased MICs, 16 to 512-fold for doxycycline, chloramphenicol or rifampicin, and 2 to 8-fold for ciprofloxacin (lowest effect against PAO509, as the MIC of ciprofloxacin was already low against this strain). Checkerboard experiments allowed to quantify interactions by FIC indexes determinations. Against PAO1, NV716 showed synergistic effects at ≥2.5 µM (Fig. 1a–c for a typical checkerboard assay). It also displayed higher degrees of synergy than PMBN when combined with the 4 antibiotics against all strains, except for ciprofloxacin against PAO1*mexAB* and PAO509. PAβN showed some degree of synergy when combined with antibiotics substrates for efflux in PAO1, PT629, and PAO1*mexAB*. No synergy was observed in combinations with alexidine, colistin or NV731 (Fig. 1d–g). NV716 at 10 µM was also synergistic with ampicillin as well as with anti-Gram-positive antibiotics like linezolid, vancomycin, or azithromycin (Fig. 1h–k).

**Fig. 2:**
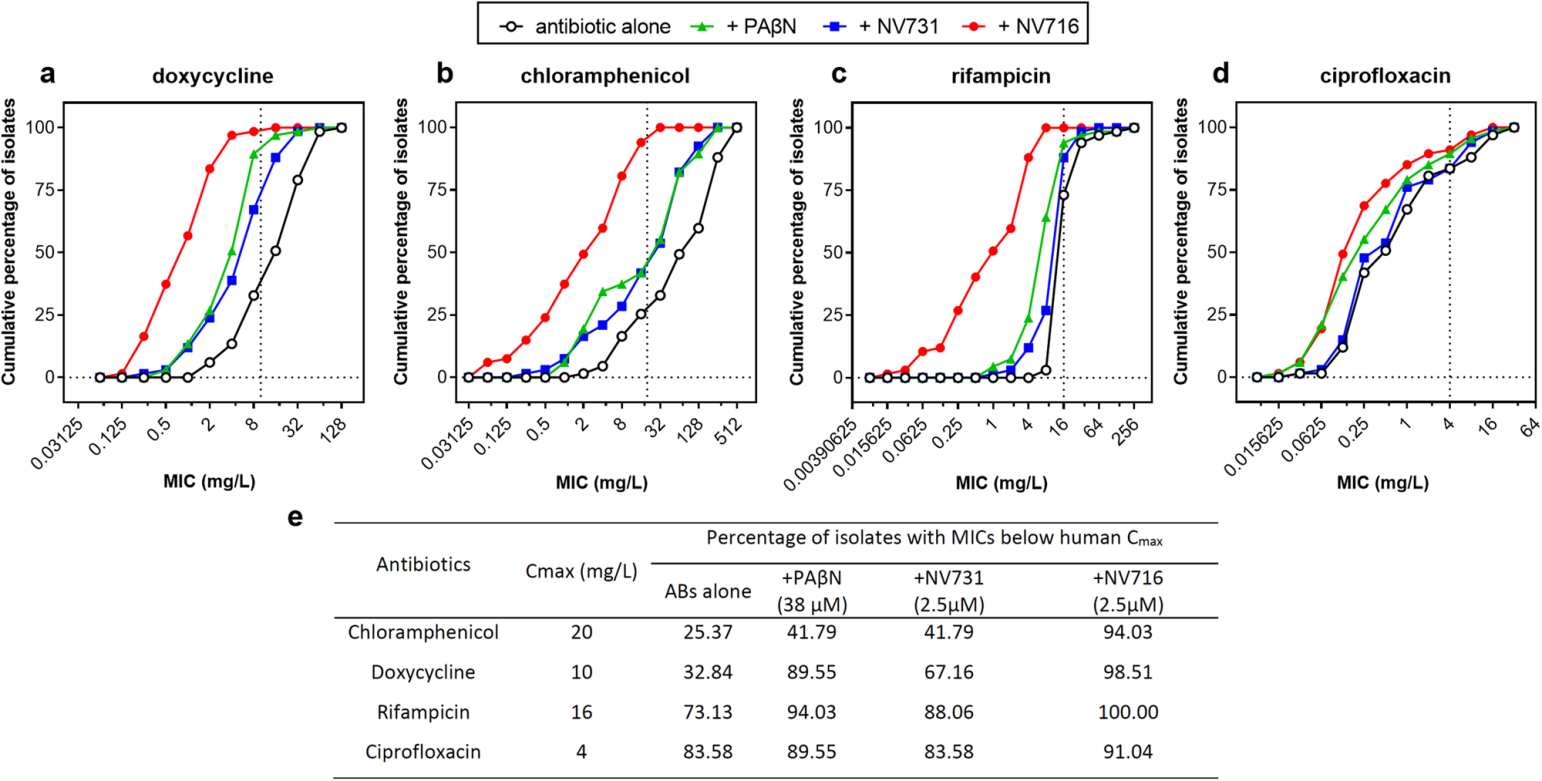
Cumulative MIC distribution for antibiotics alone or combined with potentiators (38 µM PAβN, 2.5 µM NV731 and NV716) against clinical isolates. The vertical dotted line shows the human C_max_ values for each antibiotic^75,76^ (**a** doxycycline; **b** chloramphenicol; **c** rifampicin; **d** ciprofloxacin). The table **e** shows the proportion of isolates for which MICs are below this C_max_ value, and thus falls in the range of therapeutically-achievable concentrations. All MICs were determined in at least 2-3 independent experiments (if values differing of 1–2 dilutions were obtained, the most frequent one was considered).

In clinical isolates, NV716 reduced 4 to 32-fold MIC_50_ and MIC_90_ (Supplementary Data 1) while PAβN and NV731 caused only minor changes (1–8-fold reduction). In the presence of NV716, MICs of the 4 antibiotics were in the range of therapeutically-achievable concentrations for a large proportion (>90%) of the collection (Fig. 2a–e).

The fact that NV716 increases antibiotic susceptibility, including in strains that do not express efflux (PAO509) or for antibiotics that are not substrates for efflux (rifampicin), indicates that it does not act only as an inhibitor of efflux. Its amphipathic structure suggests a possible interaction with the bacterial membrane. We therefore examined its effect in combination with the same 4 antibiotics against colistin-resistant strains (PA313, 2938, 307, 272). The LPS of these strains harbors an additional acyl chain (3-OH C10:0 or C14:0) as compared to PAO1, and, most notably, an additional 4-amino-4-deoxy-L-arabinose (L-Ara4N) cationic group (Supplementary Fig. 2), known to partially neutralize the negative charges of the outer membrane and to hinder colistin binding to LPS^23^. NV716 at 10 μM (as well as PMBN at 30 μM) remained able to decrease the MIC of antibiotics against these strains, though to a lower extent than against PAO1 (Supplementary Data 1), and to maintain synergy (Fig. 1l–o).

### Interaction with *Pseudomonas aeruginosa* membranes

**Fig. 3:**
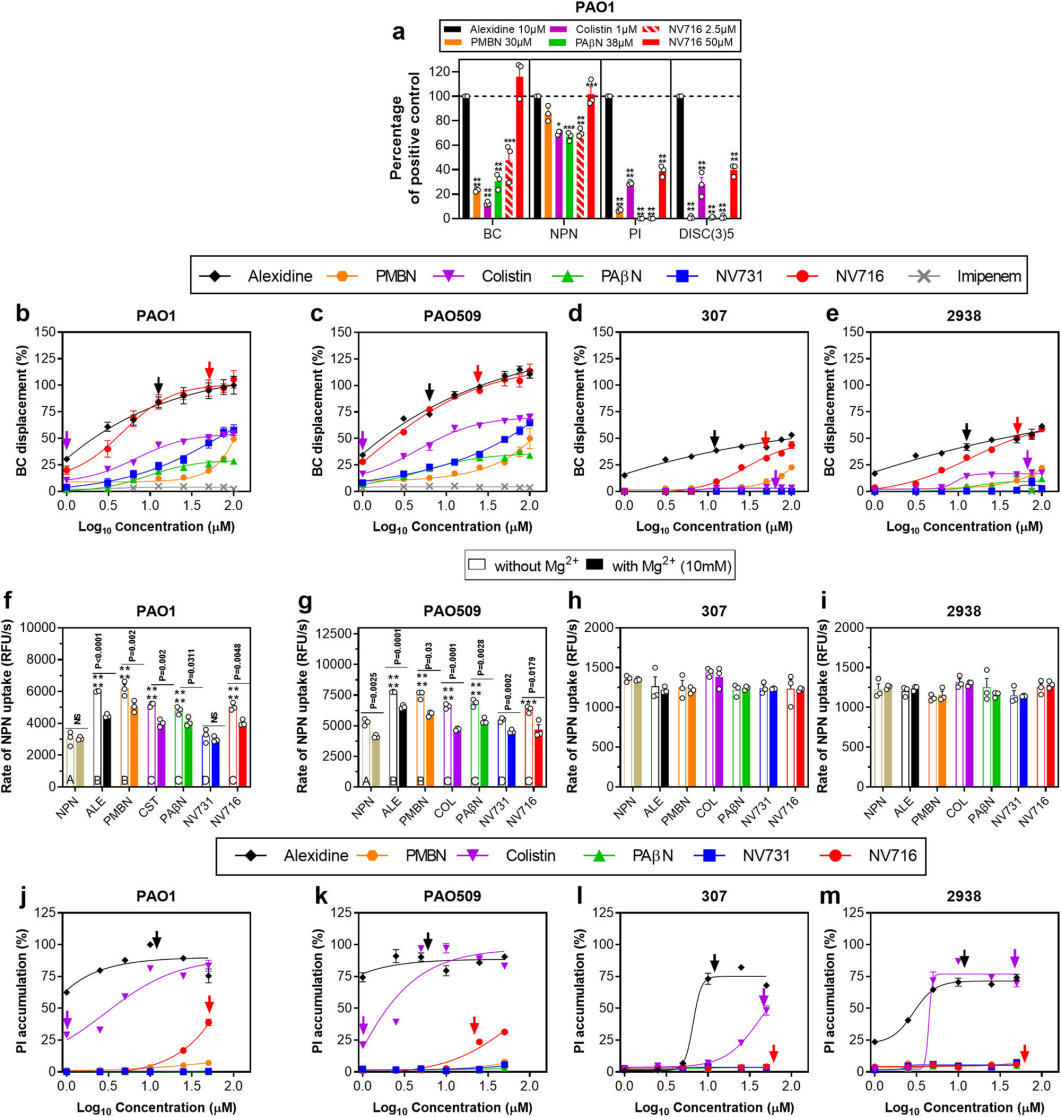
Effect of potentiators on membrane properties of the 2 reference strains PAO1 and PAO509 and 2 colistin-resistant clinical isolates PA2938 and PA307. **a** Summary of the effect of potentiators at fixed concentrations (alexidine [ALE], 2.5 µM; polymyxin B nonapeptide [PMBN]: 30 μM; colistin [CST], 1 µM (MIC against PAO1); PAβN: 38 μM; NV716: 2.5 μM and 50 µM [MIC against PAO1]) on membrane properties of PAO1 (BC: Binding to LPS, assessed by measuring the displacement of BODIPY-cadaverine after incubation during 30 min; NPN: Effect on outer membrane permeability, assessed by measuring the rate of 1-*N*-phenylnaphthylamine (NPN) uptake at early-time points (0-4 seconds); PI: Effect on inner membrane permeability, assessed by measuring the fluorescence of propidium iodide (PI) after 1 h of incubation; DISC(3)5: Inner membrane depolarization, assessed by measuring the DiSC3(5) fluorescence after 15 min of incubation. Values are expressed in percentage of the effect of alexidine. Statistical analysis: one-way ANOVA with Tukey post-hoc test: ****p < 0.0001. **b**–**e** concentration-response for BC displacement (Imipenem: negative control). **f**–**i** NPN uptake (PAβN: 38 μM; polymyxin B nonapeptide [PMBN]: 30 μM; alexidine [ALE], colistin [CST], NV731, NV716: 2.5 μM), in the absence (open bars) or in the presence (closed bars) of 10 mM Mg^2+^. Statistical analysis: one-way ANOVA with Tukey post-hoc test comparing NPN uptake in all conditions: open bars with different letters are different from one another. One-way ANOVA with Dunnett’s post-hoc test for comparison between NPN uptake alone (as a control) or combined with each of the potentiator: ***p < 0.001; ****p < 0.0001. Two-tailed Student’s *t* test for comparison for each condition without and with Mg^2+^: *p* value indicated above each pair of columns. **j**–**m** concentration-response for PI fluorescence. The effect measured with 0.5 % SDS (w/v) after 1 h was taken as 100% (positive control). All data are means ± SEM (triplicates from 3 independent experiments). In panels **b**–**e** and **j**–**m**, the arrows point to the MIC of the corresponding compound (see Supplementary Table 1; when not shown, the MIC is higher than the highest concentration tested).

We next explored the mechanism of this potentiator effect. Because NV716 is less active against colistin-resistant isolates, we examined its capacity to interact with bacterial membranes. We determined its capacity (i) to displace bodipy-cadaverine (BC) from its binding to LPS, (ii) to permeabilize the outer membrane (measure of the fluorescence of NPN when incorporated in the hydrophobic core of this membrane^24^), or (iii) to permeabilize both the outer and the inner membranes (measure of the fluorescence generated by propidium iodide (PI) when getting access to DNA), and (iv) to depolarize the inner membrane (increase in DiSC3(5) fluorescence). Alexidine and colistin were used as positive controls^20,25^ at their MIC, imipenem as a negative control (BC assay), and the other potentiators as comparators. In PAO1 (Fig. 3a), NV716 at 2.5 µM was more effective than PMBN (30 µM), colistin (1 µM; 1 × MIC) or PAβN (38 µM) to displace BC from its binding to LPS, and showed similar effects on outer membrane permeability, but no effect on inner membrane permeability or potential. At its MIC (50 µM), it was as effective as alexidine at its MIC (10 µM) in the BC and NPN assays, and as effective as colistin at its MIC in the PI and DiSC(3)5 assays.

These experiments were also performed in more diverse conditions (whole range of equimolar concentrations of potentiators for BC and PI assays; addition or not of Mg^2+^ for NPN assay) for PAO1, its mutant deleted in 5 efflux pumps PAO509 and 2 colistin-resistant isolates (PA307 and PA2938), allowing us to make additional observations (MICs of potentiators against these strains: Supplementary Table 1).

In the BC displacement assay (Fig. 3b–e), NV716 showed similar effects as alexidine over the whole range of concentrations against PAO1 and PAO509, but was less potent than alexidine against colistin-resistant isolates. The other potentiators were less effective over the whole range of concentrations and against all strains. The effects of all molecules were attenuated in a concentration-dependent fashion by the addition of Mg^2+^ (Supplementary Fig. 3).

The rate of NPN uptake was increased to a similar level by NV716 (2.5 μM), colistin (2.5 μM), and PAβN (38 µM) in PAO1 and PAO509 and to even higher levels by alexidine (2.5 μM) and PMBN (30 μM). NV731 (2.5 µM) did not show any effect. The addition of Mg^2+^ counteracted the effect of potentiators. None of the potentiators was able to increase NPN uptake in colistin-resistant isolates (Fig. 3f–i).

PI fluorescence was only modestly increased by NV716 (~50%) at high molar concentrations (50 μM) and only in PAO1 and PAO509. Alexidine, and to some extend colistin, were more effective, already at lower molar concentrations, including against colistin-resistant isolates (Fig. 3j–m). Yet, at equipotent concentrations (1 × MIC, see Supplementary Fig. 4 and arrows in Fig. 3j–m), NV716 effect was comparable to that of colistin in colistin-susceptible strains, but still lower than that of alexidine. PMBN, PAβN, and NV731 were ineffective in this assay.

### Transmission electron microscopy

To examine whether NV716 membrane effects were accompanied by visible changes in bacteria ultrastructure, we examined in electron microscopy PAO1 cells that had been exposed to a concentration of 10 µM during 1 h. As shown in Supplementary Fig. 5, bacteria maintained a morphology similar to that of control cells, with visible septa suggesting they remained capable of dividing. A massive loss of destroyed bacteria during washing procedures can be excluded, because samples exposed to NV716 did not show a reduction in their protein content (11%; SDS used as positive control). Therefore, these images suggest that NV716, at a concentration for which it proves active as a potentiator and disturbs outer membrane permeability, does not cause clear-cut alterations of bacterial integrity.

### Antibiotic accumulation

The outer membrane is a barrier to the diffusion of antibiotics. The permeabilizing effect of NV716 on the outer membrane encouraged us to examine whether potentiation of antibiotics was related to a higher antibiotic accumulation inside bacteria. We assessed therefore the effects of potentiators on the accumulation of ciprofloxacin and NV1532 (a fluorescent derivative of rifampicin), as representative of antibiotics that are, or are not, substrates for efflux, respectively.

**Fig. 4:**
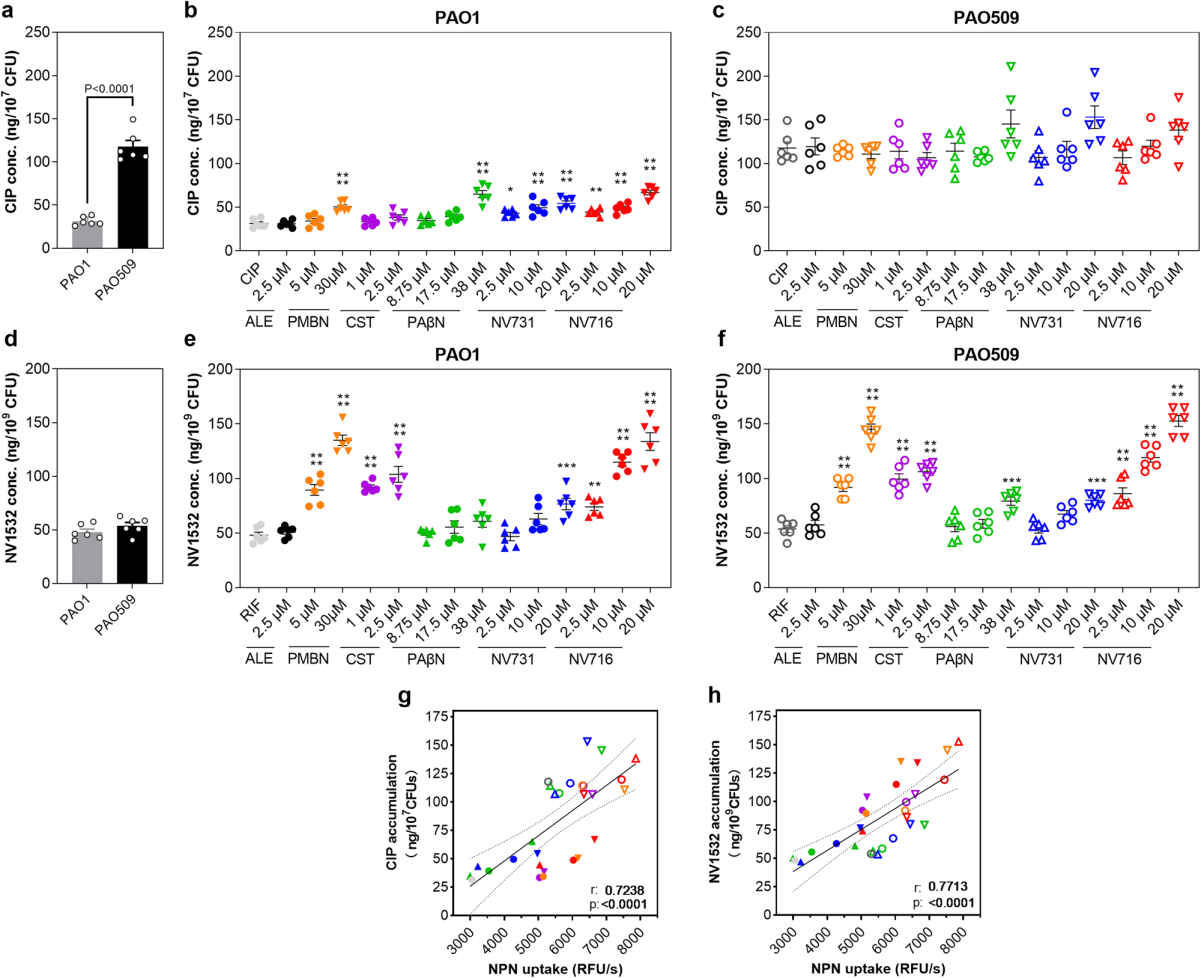
Accumulation of antibiotics in PAO1 and PAO509. Ciprofloxacin or rifampicin analog NV1532 were used alone (**a**, **d**) or combined with potentiators in PAO1 (**b**, **e**) or PAO509 (**c**, **f**). Potentiators were added at the indicated concentrations (alexidine [ALE]; polymyxin B nonapeptide [PMBN]; colistin [CST]). All data are mean ± SEM (triplicates from three independent experiments). Statistical analysis: Left-panels: Two-tail Student’s *t* test for comparison of antibiotics alone in PAO1 and PAO509; other panels: one-way ANOVA with Dunnett’s post-hoc test for comparison of antibiotics alone and combined with each potentiator: *p < 0.05; **p < 0.01; ***p < 0.001; ****p < 0.0001. Correlation between the accumulation of ciprofloxacin (**g**) or NV1532 (**h**) in bacteria and the outer membrane permeability as assessed by the fluorescence signal of NPN (see Fig. 3**f**–**i**). The Pearson correlation coefficient r and the *p* values are shown on each graph. The plain and dotted lines correspond to the linear regression with its 95% confidence interval.

As expected, the level of accumulation of ciprofloxacin was ~3 times higher in PAO509 (deleted for the expression of 5 efflux pumps) than in PAO1 (Fig. 4a). In PAO1, NV716 and NV731 increased ciprofloxacin accumulation on a concentration-dependent manner. PMBN and PAβN caused a significant increase at the highest concentration tested while alexidine and colistin did not show any effect (Fig. 4b). In PAO509, none of the compounds was able to significantly increase ciprofloxacin accumulation, even at high concentrations (Fig. 4c).

The accumulation of the rifampicin analog NV1532 (see structure in Supplementary Fig. 6) was similar in both strains (Fig. 4d). NV716, PMBN, and colistin were the only potentiators capable of causing a significant, concentration-dependent increase in its accumulation in both strains, while NV731 was only active at the highest concentration tested, and PAβN, only in PAO509 (Fig. 4e, f).

A significant correlation (Fig. 4g, h) was observed between the accumulation level of each drug inside bacteria as measured in all these conditions and the outer membrane permeability, as assessed by the fluorescence signal of NPN (data from Fig. 3f–i).

### Resistance selection

**Fig. 5:**
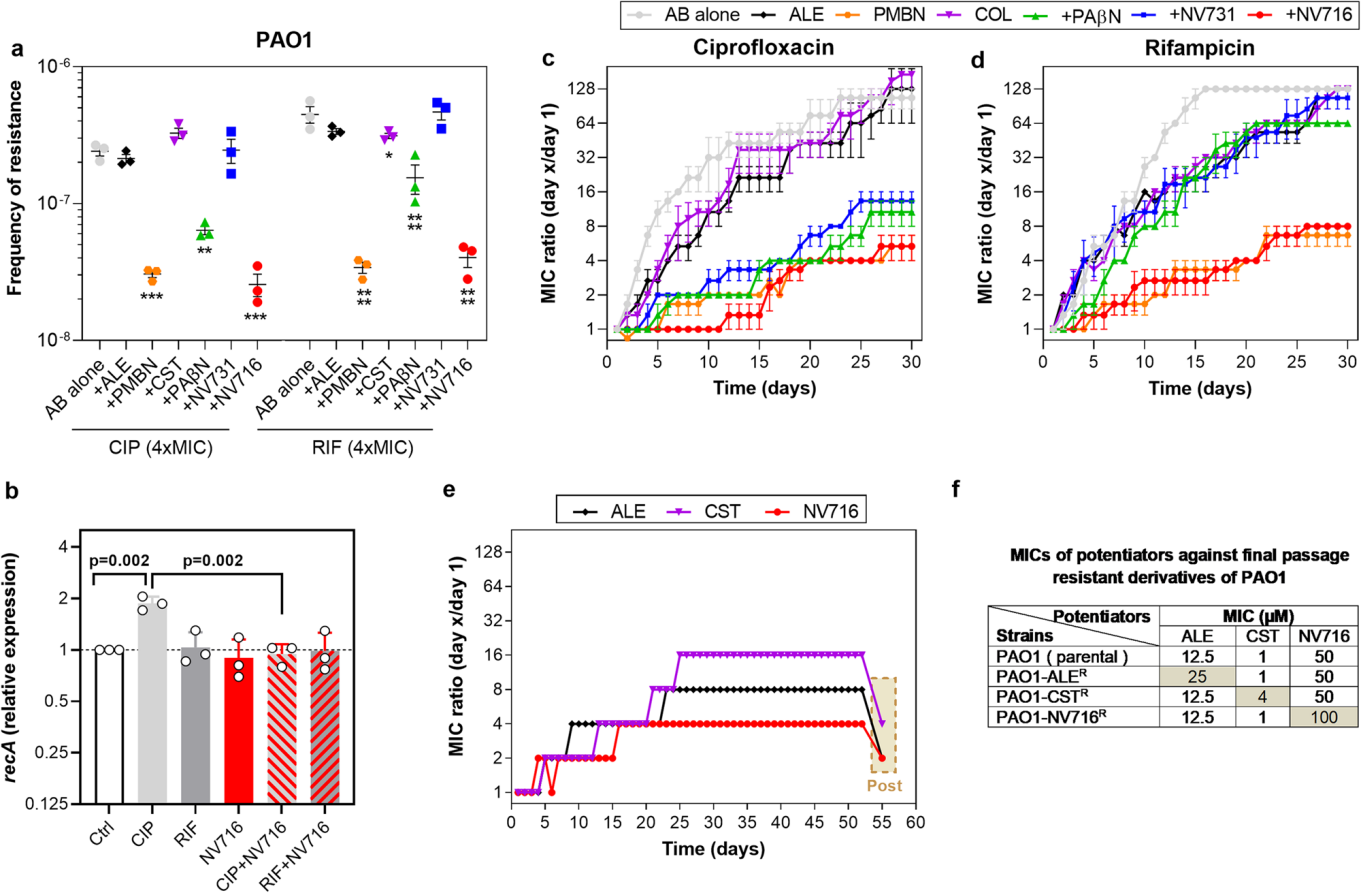
Effect of potentiators on resistance selection in PAO1. **a** Frequency of resistance to ciprofloxacin and rifampicin after 24 h of incubation with each antibiotic at 4 x MIC, in control conditions (antibiotic alone) or in the presence of alexidine (ALE, 2.5 µM), polymyxin B nonapeptide (PMBN, 30 µM), colistin (CST, 0.5 µM), PAβN (38 µM), NV731 and NV716 (2.5 µM). All data are mean ± SEM (triplicates from three independent experiments). Statistical analysis: one-way ANOVA with Dunnett’s post-hoc test comparing each combination with antibiotic alone (*p < 0.05; **p < 0.01; ***p < 0.001; ****p < 0.0001). **b** Quantitative real-time PCR of transcripts of the gene *recA* in the absence of or in the presence of antibiotics at ½ MIC, NV716 alone at 2.5 µM or their combinations. Data, expressed in fold change *vs*. control samples, are means ± SEM of three independent experiments. Statistical analysis: one-way ANOVA with Tukey post hoc test (only relevant significant differences are shown on the graph). **c**, **d** Evolution of the MIC of ciprofloxacin or rifampicin over passages in the presence of each antibiotic at ½ MIC (with daily readjustment of the concentration) alone or combined with potentiators at the same concentrations as in panel **a**. All data are mean ± SEM (duplicates from three independent experiments). **e** Evolution of the MIC of alexidine, colistin, and NV716 over passages in the presence of each antibiotic at ½ MIC (with daily readjustment of the concentration). After 52 passages, MICs were measured for all compounds after 1 culture on agar in the absence of compounds (beige box in panel **e** and values in panel **f**).

An increase in the antibiotic concentration inside bacteria could reduce the risk of resistance selection, as demonstrated for PAβN with levofloxacin in PAO1^7^. We therefore determined whether potentiators were able to prevent selection of resistance to ciprofloxacin and rifampicin. We first determined the frequency of resistance in PAO1 after 48 h of incubation with antibiotics at 4 × MIC, alone or combined with potentiators. PAO1 displayed a resistance frequency of 2.4 × 10^−7^ and 4.5 × 10^−7^ for ciprofloxacin and rifampicin, respectively. At the concentrations we used, the addition of PAβN significantly reduced the frequency of resistance to 6.4 × 10^−8^ for ciprofloxacin (4-fold decrease) and to 1.5 × 10^−7^ (3-fold decrease) for rifampicin, while NV716 as well as PMBN caused an almost 10-fold reduction in the frequency of resistance for ciprofloxacin (2.6 × 10^−8^) and rifampicin (4.0 × 10^−8^) (Fig. 5a). Alexidine and colistin had no effect at the concentration tested (lower than their respective MIC). This effect can be attributed to an increase in the accumulation of rifampicin for the three compounds, and of ciprofloxacin for PAβN and PMBN, but not for NV716, because it did not improve ciprofloxacin uptake at the concentration used here (2.5 µM; see Fig. 4b). As the development of resistance to fluoroquinolones is triggered by the SOS response^26^, we measured the expression of *recA* (derepressor of the SOS regulon) in bacteria exposed to the antibiotics and NV716 alone or in combination. As expected, ciprofloxacin (but not rifampicin) induced *recA*, but this effect was annihilated by NV716 (Fig. 5b), providing a rational explanation for its capacity to prevent resistance selection by ciprofloxacin as well.

We then evaluated the development of resistance to the same antibiotics by serial passaging over 52 days, with daily readjustment of the concentration to ½ MIC (Fig. 5c, d). In the absence of potentiator, PAO1 rapidly developed resistance to both ciprofloxacin and rifampicin, with a 128-fold increase in MIC observed after 20 and 16 passages, respectively (up to MICs of 32  and 2048 mg/L, respectively). NV716 proved highly effective in the assay, the MIC having increased only 4-16 fold at the end of the 30 days incubation when combined with ciprofloxacin and rifampicin, respectively. PAβN and NV731 only slightly delayed this selection for rifampicin while their effect remained important for ciprofloxacin, with increases in MICs of only 8-fold observed at the end of the experiment. Alexidine and colistin were largely ineffective to prevent selection of resistance to both drugs, while PMBN was highly effective.

Selection of resistance is a useful strategy to discover the targets of antimicrobial agents, as resistance acquisition is often related to genomic modifications occurring in the drug target^27^. We therefore also examined whether it was possible to select resistance to the potentiators themselves by serial passages over 52 days in control conditions or in the presence of ½ of their respective MIC, focusing on those that show low MICs (NV716, alexidine, and colistin). Resistance selection was much slower than for ciprofloxacin/rifampicin, since we observed an increase of MIC of 4–8 fold only at passage 9/23 with alexidine, 13/21 with colistin and 16/ >52 with NV716 (Fig. 5e). These resistances were also rapidly reversible, as MICs values were reduced of 1-2 dilutions after one passage culture in the absence of compound. No cross-resistance was observed between the three potentiators in the final daughter cells (Fig. 5f).

### Genomic and transcriptomic analysis and phenotypic validations

We selected three colonies of each of the passaged PAO1 and subjected the DNA to whole genome sequencing. A total of 15 SNPs, which could be unambiguously assigned to the PAO1 genome, were detected in the passaged strains (Supplementary Table 2). 5 SNPs were located in intergenic regions and 3 SNPs were synonymous mutations. The remaining 7 SNPs were found in overall 6 ORFs. In the strain treated with NV716, SNPs were found in *morA* and *roxR*. In the colistin-treated strain, we found non-synonymous mutations in *phoQ*, *aer* and *pmrB*. In the strain treated with alexidine, SNPs in *rpoB* and *roxR* (however a different position than in the NV716-treated strain) were observed. Signal transduction via PhoQ, Aer, RoxR and PmrB, has been described to be involved in resistance to polymyxins. However, the acquisition of these mutations in the strains exposed to each of the three tested agents was obviously not sufficient to induce a stable resistance phenotype against NV716, colistin or alexidine.

**Fig. 6:**
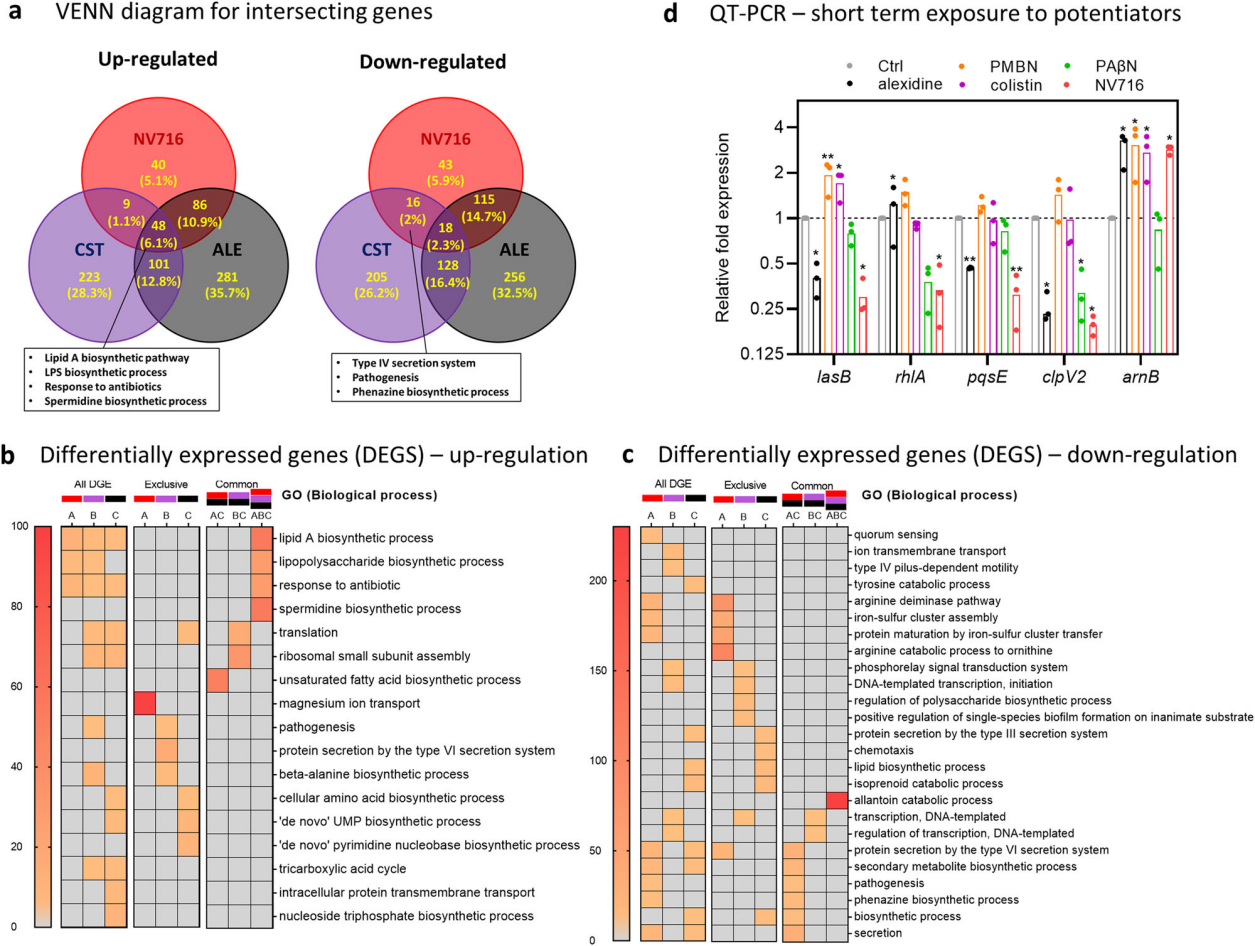
Transcriptomic analysis of PAO1 after serial passages with selected potentiators. **a** Venn diagrams showing the commonly differentially expressed genes in PAO1 after 52 passages of culture in the absence or in the presence of NV716, colistin (CST) or alexidine (ALE) at ½ MIC. Pathways for intersecting genes that are relevant for this study are depicted in boxes. **b**, **c** GO term enrichment analysis of up- and downregulated genes, respectively, highlighting the biological functions that are exclusively regulated by a single potentiator or shared by two or three of them (A, red: NV716, B, purple: colistin, C, black: alexidine). The color gradient represents the value of the fold-change enrichment. All processes shown in these panels are significant (FDR < 0.05). **d** Relative expression of genes involved in the regulation of virulence or in LPS modifications in PAO1 after 6 h of incubation in the absence (Ctrl, normalized to 1) of or in presence of potentiators at 38 µM (PAβN) or ½ MIC (other molecules). Statistical analysis: **a**: the number of up- or downregulated genes is higher than expected by chance (higher than 97.5% of permutated data) for those up- or downregulated by the three potentiators or by NV716 and alexidine. **d** One-way ANOVA with Dunnett’s post-hoc test (*p < 0.05; **p < 0.01).

Since resistance to NV716 and other potentiators was rapidly reversible, we also performed a transcriptomic analysis of the same passaged strains, reasoning that transient changes in susceptibility could be related to modifications in the expression level of genes involved in specific pathways. The number of differentially expressed genes (DEGs) between control bacteria and potentiator-exposed cells after 52 passages with NV716, colistin and alexidine was 375, 748, and 1034, respectively. They were ranked according to their false discovery rate and log_2_ fold change in Supplementary Fig. 7. Among these genes, 48 were commonly upregulated and 18 downregulated after exposure to the three potentiators (only 3 and 2 expected by chance; Fig. 6a). The upregulated genes are involved in lipid A biosynthetic process and response to antibiotics as well as LPS synthesis (Fig. 6a, b). Closer inspection of the genes that were exclusively downregulated in the NV716-treated PAO1 revealed quorum sensing and pathogenesis-related functions (Fig. 6c). Supplementary Table 3 shows in details the DEG involved in QS, as well as in elastase, rhamnolipids and pyocyanin production, all under the control of QS^28^. The most downregulated QS-related genes encode 2-heptyl-3-hydroxy-4(1H)-quinolone synthase (*pqsH*), acyl-homoserine-lactone synthase (*lasI*), acyl-homoserine-lactone synthase (*rhlI*) and the regulatory protein RsaL (*rsaL*). A selection of genes involved in elastase, rhamnolipid and pyocyanin production were also found to be downregulated in alexidine-treated PAO1 cells. To establish whether these changes were only induced after serial passages or already after short-term exposure to the potentiators, we measured the expression of typical genes involved in the regulation of the production of elastase (*lasB*), rhamnolipids (*rhlA*), pyocyanin (*pqsE*), or T6SS (*clpV2*) as well as in the remodeling of lipidA (*arnB*) in bacteria incubated with ½ MIC for 6 h. We made similar observations, namely a decrease in the expression of the 4 first genes and an overexpression *of arnB* with NV716 (Fig. 6d). A similar profile was observed with alexidine, but not with colistin (no downregulation of virulence-associated genes). These data therefore indicate that transcriptomic changes observed in passaged strains were already present after short incubation.

**Fig. 7:**
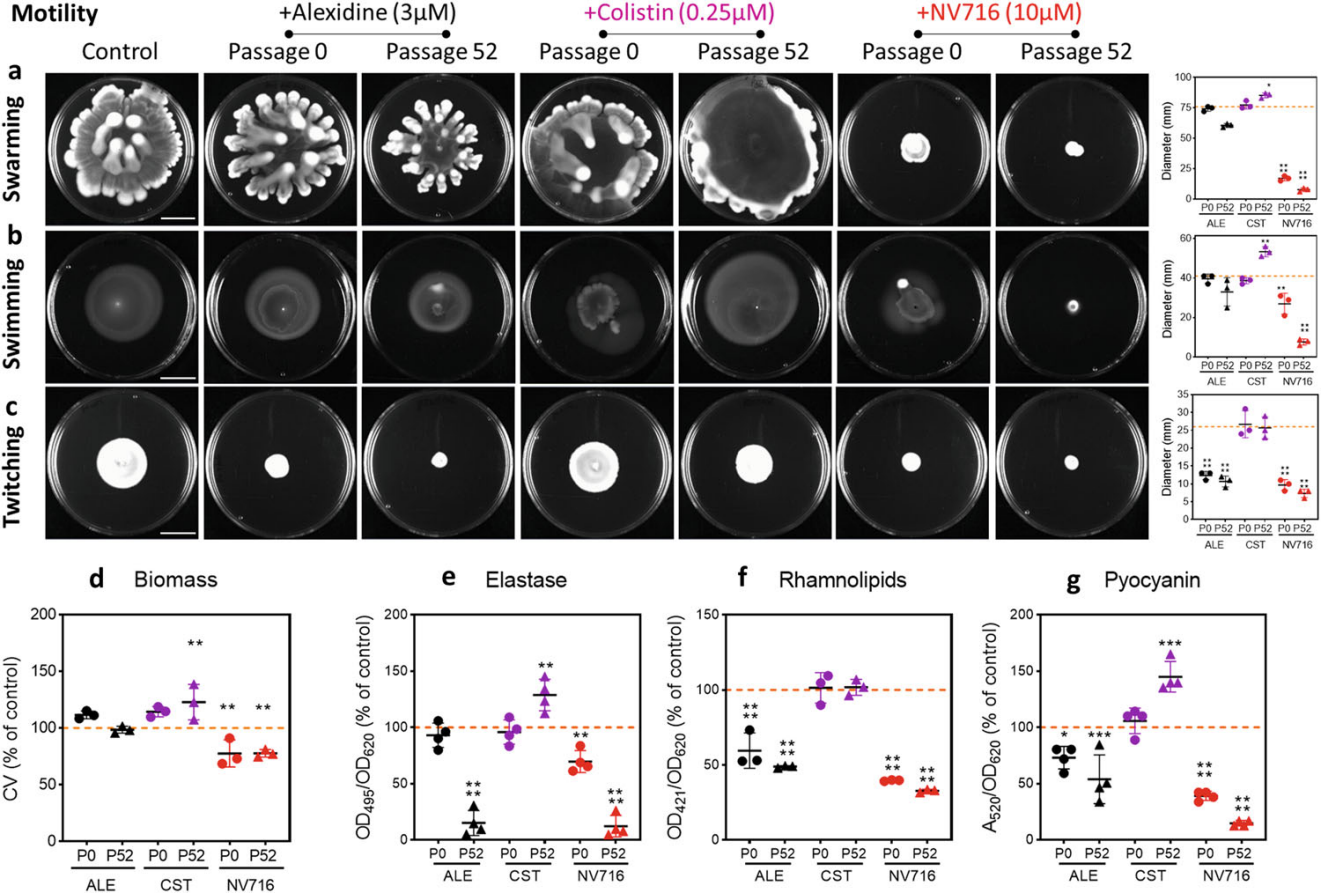
Assays validating transcriptomic analyses, performed for the parental strain or the final isolate (passage 52) exposed to each potentiator at ½ MIC. Motility assays (**a** swarming; **b** swimming; **c** twitching); scale bar: 2 cm; biofilm formation assessed by biomass quantification (**d**) and virulence factors secretion (**e**–**g**). In these panels, all data are expressed in percentage of the untreated control. Data are means of triplicates from 3 to 4 experiments. Statistical analysis: one-way ANOVA with Dunnett’s post-hoc test (*p < 0.05; **p < 0.01; ***p < 0.001; ****p < 0.0001).

To further validate these transcriptomic observations, we evaluated the effects of potentiators on motility (swarming, swimming, twitching), biofilm formation, and elastase, rhamnolipid, and pyocyanin production by comparing these parameters after exposure of the initial and the final (passage 52) bacteria (Fig. 7). NV716 was the most effective (at passage 0 [short-term incubation] and even more at passage 52) to reduce motility, biofilm formation, and virulence factors production, while alexidine reduced twitching and virulence factors secretion. Colistin rather increased swimming, biofilm biomass and production of elastase and pyocyanin.

Lastly, in order to establish whether the unanticipated reduction in virulence caused by NV716 was involved in its potentiator effects or denotated an additional action on bacterial physiology, we determined the MIC and the MBC of antibiotics in single-gene transposon insertion mutants defective in the pathways affected by NV716. As shown in Supplementary Table 4, few differences were noted, and there were in general small, suggesting that the alterations in the expression of virulence factors induced by NV716 are not (or only marginally) linked to its potentiator effects. None of these mutants showed an altered outer membrane permeability (Supplementary Fig. 8).

### Activity against biofilms

**Fig. 8:**
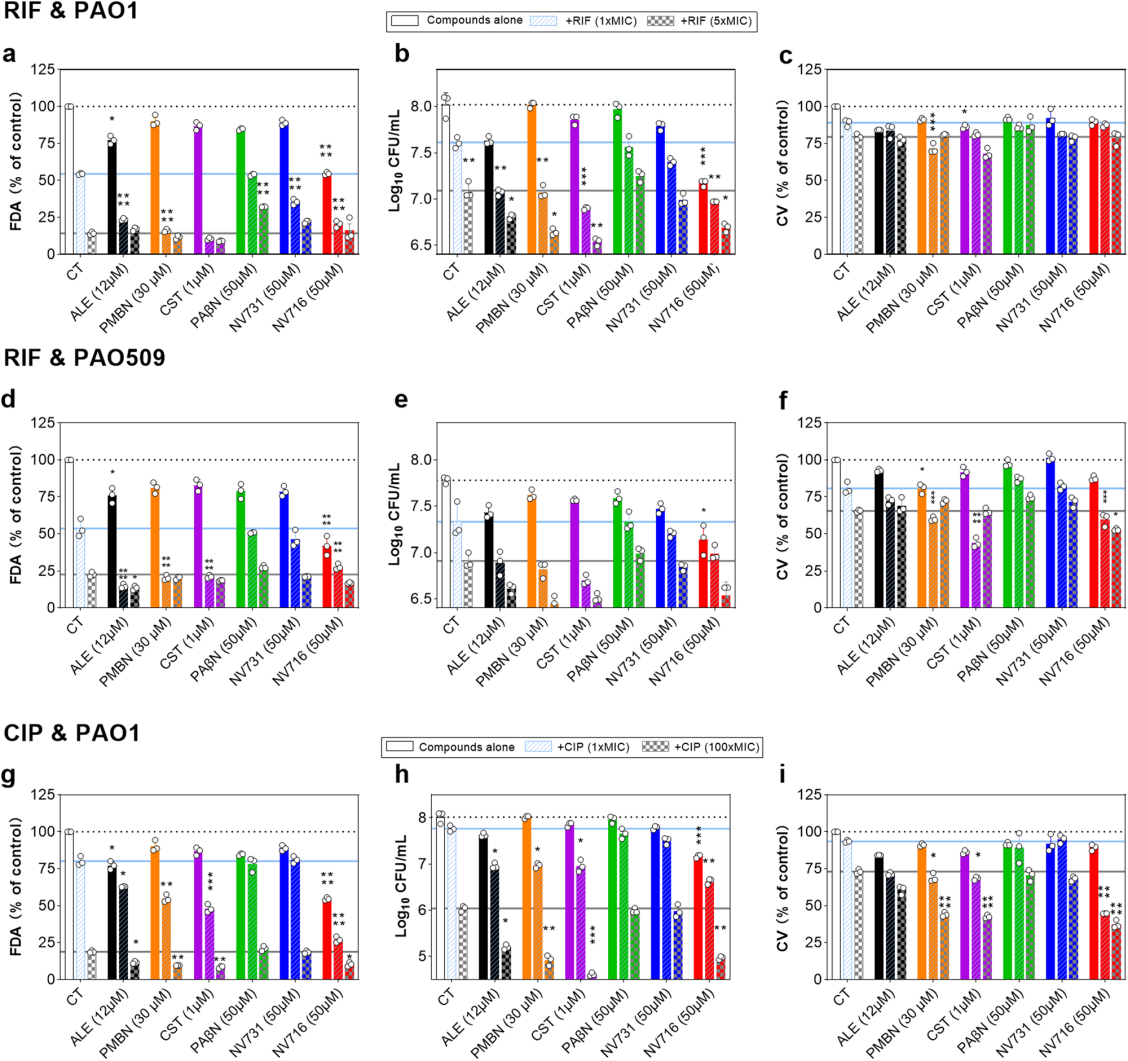
Effect of potentiators on the activity of antibiotics against biofilms. Rifampicin against biofilms of PAO1 (**a**–**c**) and PAO509 (**d**–**f**); ciprofloxacin against biofilms of PAO1 (**g**–**i**). Pre-formed biofilms were exposed during 24 h to rifampicin alone at 1 × MIC or 5 × MIC or to ciprofloxacin alone at 1 × MIC or 100 × MIC, the potentiators alone (alexidine [ALE], polymyxin B nonapeptide [PMBN], colistin [CST]) at fixed concentrations, or their combination (hatched bars: +RIF or CIP 1 × MIC; checkerboard bars: +RIF 5 × MIC or CIP 100 × MIC). The graphs show the metabolic activity (**a**, **d**, **g**; fluorescein diacetate assay [FDA]), CFUs (**b**, **e**, **h**) and biomass (**c**, **f**, **i**; crystal violet [CV] staining), respectively. All data are mean ± SEM (triplicates from three independent experiments). In each graph, the black horizontal dotted line is the value for the control (non treated biofilm), the blue line, the value for the biofilm exposed to the antibiotic alone at 1 × MIC, and the gray line, for the biofilm exposed to RIF at 5 × MIC or CIP at 100 × MIC, respectively. Statistical analysis: comparison of potentiators alone to control biofilm or to potentiator combined with rifampicin to rifampicin alone (at the same concentration) by one-way ANOVA with Dunnett’s post-hoc test: *p < 0.05; **p < 0.01; ***p < 0.001; ****p < 0.0001.

Biofilms are tolerant to antibiotic treatments, as they oppose a barrier to the penetration of antibiotics and they host metabolically less active bacteria. Noteworthy, the matrix of biofilms of Gram-negative bacteria are predominantly composed of extracellular polymeric substances (EPS) that are negatively-charged, allowing association with divalent cations to improve biofilm stability^29^. A similar type of bridging is described between Mg^2+^ and LPS. Based on our observation that NV716 can displace Mg^2+^ from LPS, we speculated that it might also impact antibiotic activity against biofilms. We therefore examined the activity of ciprofloxacin (Fig. 8a–f) or rifampicin (Fig. 8g–i) alone or combined with NV716 or the other potentiators against biofilms made by PAO1 and PAO509. Considering first the effects on metabolic activity, NV716, and to some extent, alexidine were the only potentiators capable of reducing the fluorescence signal emitted by fluorescein, the metabolite of fluorescein diacetate produced by metabolically active bacteria (Fig. 8a, d, g). This effect can be interpreted as denoting a reduction in the number of viable bacteria rather than in the intrinsic metabolic activity of bacteria, as NV716 did not decrease, but rather increased, fluorescein diacetate metabolization in planktonic cultures, probably as a consequence of an increase uptake of the dye in the cells (Supplementary Fig. 9). This reduction in bacterial load could be related to the capacity of NV716 to reduce the fraction of persisters selected by both antibiotics in stationary phase cultures (Supplementary Fig. 10). The other potentiators also improved antibiotic activity at 1 × MIC with the noticeable exception of PAβN. Changes in CFU counts were in general parallel to those in metabolic activity (Fig. 8b, e, h). None of the potentiators was capable of reducing biofilm biomass when used alone (Fig. 8c, f, i) but NV716, PMBN, and colistin improved the effects of rifampicin against PAO509 biomass and of ciprofloxacin against PAO1 biomass.

**Fig. 9:**
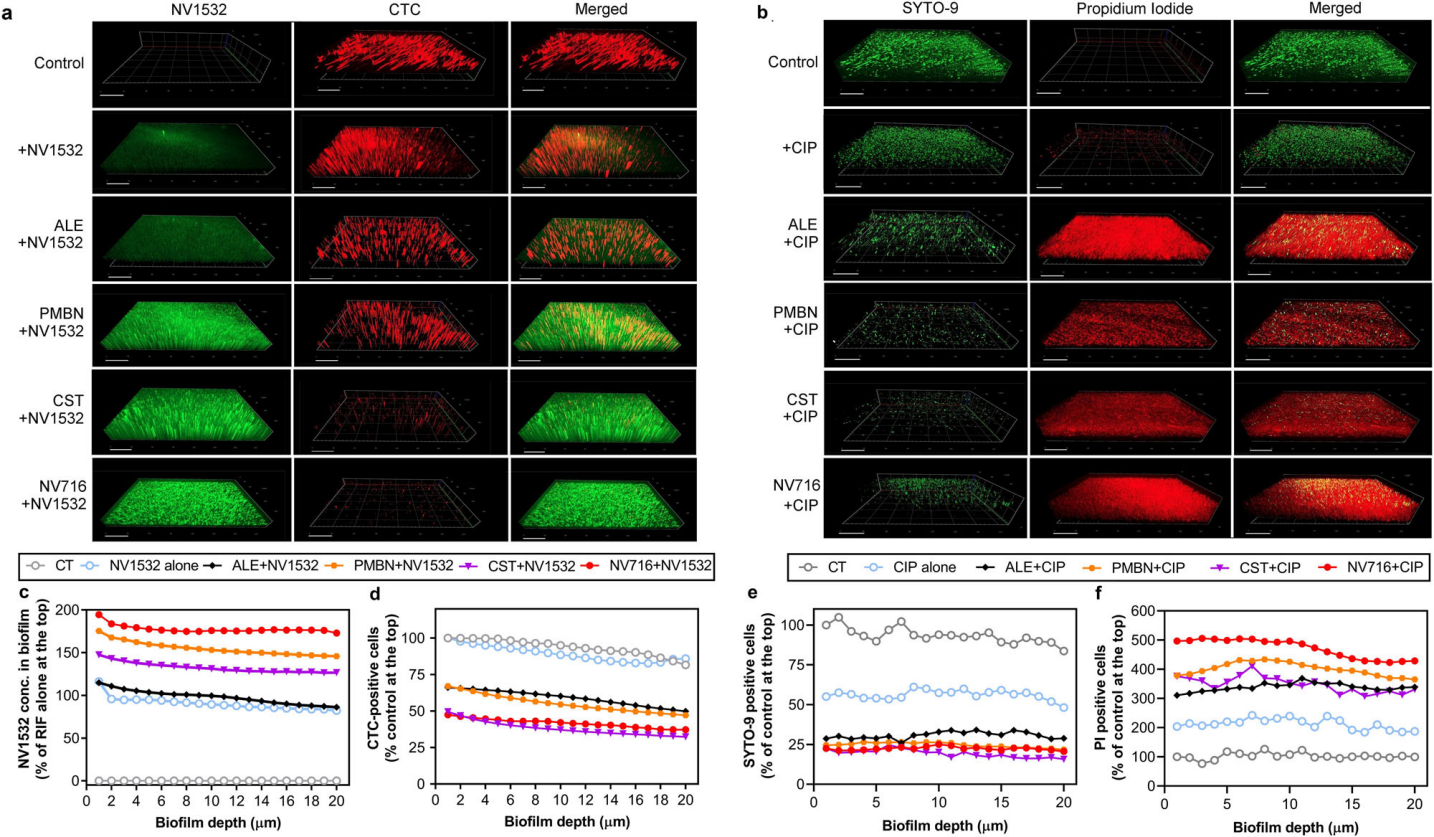
Confocal images of biofilms of PAO1 exposed to antibiotics and potentiators. Potentiators were used in the conditions described in Fig. 8 (alexidine [ALE, 12 µM], polymyxin B nonapeptide [PMBN, 30 µM], colistin [CST, 1 µM]; NV716 50 µM). **a** Biofilms incubated with NV1532 (fluorescent derivative of rifampicin) at 100 mg/L alone or with potentiators, with fluorescence of NV1532 recorded in the green channel and fluorescence of CTC in the red channel. **b** Biofilms incubated with ciprofloxacin at 50 mg/L alone or with potentiators, with fluorescence of SYTO-9 recorded in the green channel and fluorescence of propidium iodide (PI) in the red channel. Scale bar: 30 µm. **c**, **d** quantitative analysis of the data from panel **a**, showing the fluorescence of NV1532 or of CTC, respectively, in percentage of the value measured at the top of the biofilm. **e**, **f** quantitative analysis of the data from panel **b**, showing the fluorescence of SYTO-9 or of PI, respectively, in percentage of the value measured at the top of the biofilm.

Biofilms of PAO1 were then observed in confocal microscopy to visualize the penetration of the fluorescent derivative of rifampicin NV1532 in parallel with live cells (Fig. 9a, b). Colistin, PMBN, and NV716 (in that order) increased the penetration of NV1532 and the four potentiators tested increased its effect on viability (Fig. 9c, d). When combined with ciprofloxacin, all potentiators allowed to observe a marked increase in the proportion of dead cells, NV716 being the more effective (Fig. 9e, f). These data are coherent with quantitative data from Fig. 8.

## Discussion

**Fig. 10:**
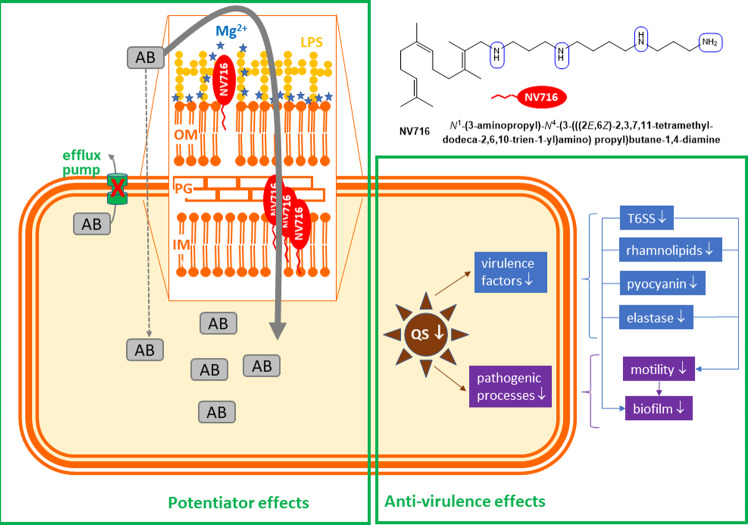
Model describing the putative mode of action of NV716 as an adjuvant molecule capable of potentiating antibiotic activity and of exerting anti-virulence effects at sub-MIC concentrations. (1) Potentiator of antibiotic activity: At subMIC concentration, NV716 interacts with LPS and increases the permeability of the outer membrane of *P. aeruginosa*, while at the same time inhibiting the activity of efflux transporters by an unknown mechanism^13^, which facilitates the penetration of antibiotics. At its MIC, it also permeabilizes the inner membrane of the bacteria, leading to their death without gross alteration of the bacterial morphological integrity. (2) Anti-virulence compound: It also reduces the expression of quorum sensing-related genes, causing a downregulation of virulence factors like rhamnolipids, pyocyanin, elastase, and of T6SS-related genes, as well as a subsequent reduction in motility and biofilm formation. The chemical structure of NV716 is also shown, with the aminated functions (protonable at physiological pH) circled in blue.

The present study demonstrates that the polyaminoisoprenyl compound NV716 is a potentially useful adjuvant with dual promising activities on *P. aeruginosa*. First, it is a potent potentiator of poorly permeable antibiotics in *P. aeruginosa*. At subMIC concentration (0.1-fold MIC), it proves capable of markedly decreasing the MIC of several of these antibiotics. This effect is at least partially independent of its previously demonstrated capacity to inhibit the activity of efflux transporters^13^, as it is also efficient against strains that do not express efflux pumps or for antibiotics that are not substrates for efflux. Importantly, NV716 does not easily select for resistance, essentially causing reversible transcriptomic changes after prolonged exposure; it also prevents selection of resistance to antibiotics, which are clear advantages for a molecule directed towards a multidrug-resistant pathogen like *P. aeruginosa*. Lastly, its potentiator effect is observed in models of difficult-to-treat infections like intracellular infection^15^ or biofilms, which broadens the spectrum of infections where it could prove useful. Second, independently of these potentiator effects on antibiotic activity, NV716 also behaves as an anti-virulence compound, decreasing the expression of quorum-sensing, with subsequent reduction in virulence, biofilm formation, and motility. Our current understanding of its dual mode of action is depicted in Fig. 10.

Intriguingly, the mechanism of action of NV716 as a potentiator shares some similarities but also notable differences with that of colistin, as well as with that of alexidine and PMBN. NV716 shares with alexidine the highest potency to bind to LPS and permeabilize the outer membrane, but, in contrast to NV716, alexidine has only minor effects on antibiotics MIC and uptake inside bacteria. The 3D molecular model of alexidine and NV716 suggests a similar, extended conformation of their positively charged moiety, which may explain this privileged interaction with LPS (see Supplementary Figs. 11 and 12). Conversely, PMBN shows similar effects as NV716 on MICs but less on displacement of bodipy-cadaverine from LPS and on the accumulation of antibiotics that are substrates for efflux. PMBN and alexidine differ in their mode of interaction with LPS. While the small lipophilic tail of PMBN is critical for its interaction with the outer membrane^30^, one of the guanidinium groups in alexidine associates with the negatively-charged phosphate group on lipid A, while the other symmetrical end of the molecule is closely associated with the phosphate group of the core portion of LPS^21^. This therefore suggests that an interaction with the lipophilic core of the membrane, which does not occur with alexidine, is needed to allow antibiotic permeation. Conversely, colistin, with a bulky charged cyclic peptide and a short lipophilic tail, is as potent as NV716 in terms of outer membrane and inner membrane permeation, the first being obtained at equimolar concentrations, and the latter, at their respective MIC. However, NV716 can increase antibiotic accumulation at subMIC concentrations (i.e., when it permeabilizes only the outer membrane), which is not the case for colistin. Studies on outer membrane models, planar bilayers, or using molecular modeling suggest that colistin interacts with the headgroups of the lipids in the outer membrane, and causes their reorientation and local disorganization as well as the formation of LPS clusters, which promotes outer membrane destabilization and gives access to colistin or other antibiotics into the periplasmic space^31–33^. Our electron microscopic images suggest that the permeation effects of NV716 occur without gross disruption of the bacterial envelope, which is coherent with the fact they occur at subMIC concentrations. Our study was not powered to perform in-depth structure-activity relationships, but encourages further efforts with homogeneous series of compounds in order to better understand these interactions at the molecular level. In this context, the comparison of the physicochemical properties of the potentiators used in this study is instructive (Supplementary Table 5). On the one hand, alexidine and NV716 show similar calculated logD values at pH 7.4, as well as similar van der Waals surface and volume, which may therefore be determinant in their capacity to interact with LPS. On the other hand, NV716 and NV731 are the only molecules showing a calculated polar surface area (PSA; defined as the surface sum over all polar atoms or molecules, primarily oxygen and nitrogen) smaller than 140 Å^2^. This parameter is a commonly-used medicinal chemistry metric to optimize the ability of a drug to cross biological barriers. A value higher than this threshold predicts a poor permeation, at least across the blood-brain barrier^34^. Whether is also applies to bacterial cells is unknown, but this exclusive property may contribute to explain the unique combination of actions shown for NV716 as compared to other potentiators. In addition, the strong difference in the spatial arrangement of the two polyaminoisoprenyl compounds (Supplementary Fig. 11), globular for NV731 (only two nitrogen groups able to interact at the same time with the negatively-charged groups of the membrane) but linear for the spermine moiety of NV716 (all aminogroups capable of interaction with the negative charges) could explain the much higher potency of NV716 in all our assays.

The global similarity in the targets of NV716, colistin, and alexidine is confirmed by our transcriptomic analysis, which shows that these molecules can upregulate the metabolic pathways involved in the synthesis of lipid A and of LPS. These pathways are known to be affected in stable mutants resistant to polymyxins^23,35^. Interestingly enough, the present work also reveals downregulation of quorum-sensing-related genes and functions that were phenotypically confirmed by a reduction in the secreted amounts rhamnolipids, pyocyanin or elastase, of biofilm formation and of motility, all under the control of the three closely interconnected Rhl, Las, and PQS systems^36^. The downregulated virulence factors themselves also contribute to reduce biofilm formation, notably by impairing motility for rhamnolipids as well as for T6SS, by activating the nucleoside diphosphate kinase for elastase, or by favoring the release of extracellular DNA for pyocyanin^37,38^. The mechanism by which NV716 perturbs quorum sensing is unknown, but probably not directly related to outer membrane permeation (as it is not observed with PMBN), neither to its potentiator effects. It remains, however that these anti-virulence properties confer to NV716 a unique profile to fight against *P. aeruginosa* infection.

Other types of membrane-active agents have been described in the literature as potentially useful alternatives for the treatment of Gram-negative multidrug resistant bacteria. These include notably a series of amphipathic antimicrobial peptides (see refs. ^39–41^ for a few recent examples). We notice however some drawbacks for these peptides as compared to NV716. First, their peptidic nature makes them more expensive to produce or to isolate from natural sources and confers to them unfavorable pharmacokinetic properties^42^. Second, many of them are hemolytic or cytotoxic, sensitive to hydrolysis by proteases, or inactivated by salt or serum^42^. Third, their proteic nature raises concern regarding a potential immunogenicity^43^. Forth, they usually act as antibiotics rather than as potentiators, which is more likely to lead to resistance^44^. This risk has been demonstrated for the AA139 derivative of the natural arenicin-3, which selects for mutations in *mlaC*, a phospholipid transport gene^41^, or for polymyxins, which induce modifications in LPS or lipid A^23^. Non peptidic membrane-permeabilizing antibiotics have also been developed, which do not fully alleviate the risk of resistance development or of cytotoxicity, as shown for 1-((2,4-dichlorophenethyl)amino)-3-phenoxypropan-2-ol (SPI009) or amphiphilic derivatives of aminoglycosides^45,46^. Likewise, many membrane-active compounds isolated from plants, like triterpenes, lack of selectivity and are cytotoxic^47^.

Although we did not uncover the precise molecular target of NV716 in *P. aeruginosa*, we nevertheless progressed in the elucidation of the physiological processes it can affect in bacteria as well as in the demonstration of their consequences for antibiotic activity and bacterial virulence. The promising profile of activity we evidenced here already at sub-inhibitory concentrations, coupled with its previously demonstrated lack of toxicity in vitro at microbiologically active concentrations^13,15^, encourages further research in pertinent in-vivo models of infections in order to better delineate its potential as an adjuvant to current antibiotics. In a broader context, this works confirms that membrane-targeting approaches are promising strategies that can revive antibiotic activity, including against persistent forms of infections like biofilms or intracellular survival.

## Methods

### Bacterial strains and culture media

Four reference strains (PAO1^48^, PT629 [MexAB overproducer derivative of PAO1^49^], PAO1mexAB^50^, PAO509 [PAO1 Δ(mexAB-oprM) Δ(mexCD-oprJ) Δ(mexEF-oprN) Δ(mexJK)^50^]) and 67 clinical isolates (Supplementary Data 1) obtained from patients with cystic fibrosis (*n* = 43), urinary tract infections (*n* = 10), or hospital-acquired pneumonia (*n* = 14) were used in this study. Transposon mutants were obtained from the PAO1 library of the University of Washington^51^. For specific experiments, additional clinical isolates resistant to colistin^25^ were also included. Bacteria were inoculated overnight at 37 °C in tryptic soy agar (VWR). A single colony was then added to 10 mL cation-adjusted Mueller-Hinton Broth (CA-MHB; Sigma-Aldrich) and incubated at 37 °C overnight under gentle agitation (130 rpm). CFUs (colony-forming units) were counted on Tryptic soy agar.

### Antibiotics and adjuvant compounds

Antibiotics (potency) were obtained as microbiological standard from Sigma-Aldrich (Chloramphenicol [98%], doxycycline [98%], rifampicin [98%], colistin [92%], alexidine [98%]) or Bayer (Leverkusen, Germany) (ciprofloxacin HCl [89%]). The reference efflux pump inhibitor Phe-Arg-β-Naphthylamide (PAβN; potency, 98%) and the membrane permeabilizer polymyxin B nonapeptide (PMBN) were purchased from Sigma-Aldrich. The hydrosoluble hydrochloride polyamino-isoprenic salt derivatives, NV716 and NV731, were synthesized at Aix-Marseille University^52^. Supplementary Fig. 1 shows the structure of the potentiators. Stock solutions were prepared in DMSO at a concentration of 96 mM [50 mg/mL] for PAβN or in water at a concentration of 10 mM for PMBN [10 mg/mL], NV716 [4 mg/mL] and NV731 [3.2 mg/mL], and stored at −20 °C until use.

### MIC and MBC determination for antibiotics alone or combined with adjuvants

MICs were determined by microdilution in CA-MHB, following the guidelines of the Clinical and Laboratory Standards Institute. After 18–20 h of incubation, the MICs were defined as the minimal concentrations preventing visible growth. MBC were calculated as the lowest antibiotic concentration reducing of 99.9% the bacterial number in aliquots from wells were no growth was observed in MIC determinations, as determined by CFU counting on agar containing 2 g/L charcoal.

### Checkerboard assay

Checkerboard assays were performed in 96-wells plates. Compound A was serially diluted from columns 1–11, and compound B, from rows B to H, with serial dilutions of compound A alone found in row A and of compound B alone in column 12. MICs of compounds alone and in combinations were used to calculate the fractional inhibitory concentration (FIC) index (FICI), as follows: ${{{{{\rm{FICI}}}}}}=\frac{{C}_{A}}{MI{C}_{A}}+\frac{{C}_{B}}{MI{C}_{B}}$, where MIC_A_ and MIC_B_ are the MICs of compounds A and B alone, and C_A_ and C_B_ are the MIC of compounds A or B in combination. The combination was considered synergistic when the FICI was <0.5, and antagonistic when the FICI was >4^53^.

### BODIPY™-TR-cadaverine displacement assay

Binding affinity of potentiators to LPS was investigated using the BODIPY™-TR-cadaverine (BC) displacement assay. When bound to LPS, BC is weakly fluorescent (self-quenching) but its fluorescence is increased when competitively displaced by other compounds displaying affinity for LPS (dequenching). As previously described^54^, BC (Thermo Fisher Scientific; final concentration, 5 μM) and bacteria (final OD_620nm_, 0.1) were mixed, kept for 30 min in the dark at room temperature, after which 50 µL of this mixture was incubated with 50 μL of test compounds in 96-wells black plates during 30 min before fluorescence measurements.

### Outer membrane permeability assay

Outer membrane permeability was assessed using the 1-*N*-phenylnaphthylamine (NPN) uptake assay^25^. The fluorescence of this probe increases when incorporated in the hydrophobic core of a permeabilized outer membrane. Concisely, 20 μL of NPN solution (final concentration, 10 μM) containing or not the test compounds were mixed with 180 μL of bacteria suspension (final OD_620nm_ = 0.5, resuspended in phosphate buffer [NaCl 110 mM; KCl 7 mM; NH_4_Cl 40 mM; NA_2_HPO_4_ 0.4 mM; Tris base 62 mM; Glucose 0.2%; pH 7.5 adjusted with HCl]) in 96-wells plates. The fluorescence was rapidly measured using a Synergy H1 Hybrid Multi-Mode Microplate Reader (BioTek) with 4 s intervals during 5 min (λexc/λem: 340/410 nm). Preliminary experiments showed that NPN uptake proceeded following a 1-phase association, with a first rapid and almost linear uptake during the first four seconds, followed by a plateau (Supplementary Fig. 13). As NPN is a substrate for efflux in *P. aeruginosa*^55^, we calculated the rate of its influx for the early time points (0–4 seconds) to minimize a possible contribution of efflux in the reduction of its rate of uptake, assuming a linear uptake over this period of time and report these data.

### Inner membrane permeability assay

Inner membrane permeability was determined using the Propidium Iodide (PI) assay as previously described^56^. PI becomes fluorescent when intercalated in the DNA of bacteria, the outer and inner membrane of which are permeabilized. Briefly, overnight cultures were centrifuged and resuspended in phosphate buffer (same composition as above) to reach an OD_620nm_ of 0.1. Five μL PI (final concentration, 6 μM) was added to 5 mL of bacterial suspension and the mixture was incubated during 30 min in the dark. Fifty μL of this mixture were added by 50 µL of test compounds in a 96-wells plate. After 60 min incubation at room temperature, the fluorescence of PI was measured (λexc/λem: 535/617 nm) in a SpectraMax M3 plate reader.

### Inner membrane depolarization assay

We used the previously described DiSC3(5) (3, 3-dipropylthiadicarbocyanine iodide) assay^13^. Overnight cultures were pelleted by centrifugation (3000 × *g*, 7 min), washed (1× PBS) and resuspended with 50 mM Tris buffer saline (pH 7.4) containing 2 mM EDTA. After 5 min of incubation, cells were pelleted (3000 × *g*, 7 min), washed in PBS, resuspended with Tris buffer saline containing 50 mM glucose and adjusted to obtain an OD_620nm_ of 0.5, after which DiSC3(5) (final concentration of 10 μM) was added. 100 μL of potentiator were mixed with 100 μL bacteria suspension containing DiSC3(5) in a 96-well plate. After 30 min of incubation, the fluorescent intensity was measured in a Spectramax M3 plate reader (λexc/λem: 622/670 nm). Alexidine at 10 µM was used as a positive control and cells added by Tris buffer saline as a negative control.

### Antibiotic accumulation in bacteria

Ciprofloxacin accumulation inside bacteria was determined by a fluorometric method^57^. Briefly, 10 mL of bacterial suspension (~10^9^ cells/mL) were incubated with ciprofloxacin (final concentration, 10 mg/L) with or without potentiators at selected concentrations during 15 min (i.e., a duration longer than the predicted time required to reach saturation^58^, but during which the antibiotic did not affect bacterial growth [no significant reduction in CFUs]). After appropriate dilution in PBS, 50 µL aliquots were spread on TSA and colony-forming units (CFUs) were counted after overnight incubation at 37 °C. The pellet was then collected by centrifugation at 3000 × *g* for 7 min at 4 °C, washed 3 times in cold PBS to remove ciprofloxacin from the medium and then resuspended in 0.1 M glycine-HCl buffer (pH 3). Samples were kept in the dark overnight to allow bacteria lysis and ciprofloxacin release. After centrifugation at 20,000 × *g* for 7 min, ciprofloxacin fluorescence was measured in the supernatant (λexc/λem: 275/450 nm) in a Spectramax M3 plate reader. Ciprofloxacin concentrations were normalized to CFUs counts.

Rifampicin accumulation was measured using a fluorescent derivative (NV1532, see structure in Supplementary Fig. 6) synthesized by one of us (JMBr; see Supplementary Method S1 and Supplementary Scheme 1). This compound remained microbiologically active, with a MIC of 32 mg/L against PAO1 (*vs*. 16 mg/L for rifampicin). The fluorescence emission peak of NV1532 in PBS was determined by an emission scan at an excitation wavelength of 470 nm. The lower limit of detection and of linearity of the fluorescence calibration curve were 15.6–1000 μg/L (Supplementary Fig. 14). In brief, 10 mL of bacterial suspension (~10^9^ cells/mL) were incubated for 15 min with NV1532 (final concentration, 20 mg/L) combined or not with potentiators. Aliquots were processed for CFU counts and the rest of the samples were centrifuged, washed to remove NV1532 as described for ciprofloxacin assay, resuspended in 500 μL PBS and sonicated 60 min in a bath to lyse the bacteria. After centrifugation, the supernatant was used to measure fluorescence (λexc/λem: 470/525 nm) in a Spectramax M3 plate reader and data normalized to CFU counts.

### Frequency of selection of resistance and selection of resistance by serial passages

PAO1 (10^9^ CFU/mL) was spread on TSA plates containing ciprofloxacin or rifampicin at a final concentration of 4 × MIC alone or combined with potentiators. The frequency of selection of antibiotic-resistant mutants was determined as the ratio between the number of colonies that appeared after 48 h of incubation at 37 °C on the drug-containing plates and control plates^59^. MIC increase was also followed over serial passage cultures in broth exposed to (i) ¼ MIC of the antibiotic alone or combined with potentiators or (ii) potentiators alone at ½ MIC, with daily 1/1000 dilution of the bacteria and readjustment of the concentration to (i) ¼ MIC or (ii) ½ MIC of the previous day, over approx. (i) 30 or (ii) 50 days.

### Genomic and transcriptomic analysis

PAO1 and daughter bacteria collected after the 52^th^ passage in the presence of potentiators at ½ MIC were spread on TSA containing or not potentiators at ½ MIC and incubated overnight, after which 3 colonies were independently inoculated in 2 mL MHB-CA containing or not ½ MIC of potentiators and incubated overnight at 37 °C with shaking at 130 rpm.

DNA was extracted from cell pellets using the DNeasy Blood & Tissue Kit (Qiagen) and 0.8 ng/μL DNA was used for the library preparation based on a modified version of the Illumina Nextera XT protocol with unique dual indices. The library pool was size selected (500–1500 bp) on the BluePippin™ (Sage Science) instrument. Libraries were sequenced in paired-end mode on an Illumina NovaSeq 6000 device (2 × 50 bp). The obtained reads were quality controlled by fastqc with default settings^60^. The tool suite Bbtools was used for cleaning and merging reads, and for performing statistical analyses on coverage and insert sizes (BBMap – Bushnell B. – sourceforge.net/projects/bbmap/). The trimmed reads were de-novo assembled using Shovill (https://github.com/tseemann/shovill) with the assembler SPAdes with the optional setting ‘only-assembler’. Single nucleotide polymorphisms (SNPs) were detected by Parsnp^61^ with default settings and with PAO1 as the reference. The raw sequencing files have been deposited in the NCBI SRA database under the accession number PRJNA785668.

RNA was extracted from cell pellets using the InviTrap® Spin Cell RNA Mini Kit (INVITE). Ribosomal RNA was removed using the Ribo-Zero Bacteria Kit (Illumina) and libraries for transcriptomics were generated according to references^62,63^. Libraries were sequenced in paired-end mode on an Illumina NovaSeq 6000 device (2 × 50 bp). The obtained reads were quality controlled (option ‘—nextseq-trim=20’) and clipped using cutadapt^64^. Reads were mapped to the reference genome PAO1 (NC_002516.2) using bowtie2^65^. The resulting sam-files were converted to indexed binary format using SAMtools^66^. Counts of the mapped reads were extracted with featureCounts^67^ using reads with a minimum mapping quality of 20. Differential gene expression analysis was performed with the R package edgeR^68^. Normalization factors to scale the raw library sizes were calculated using the weighted trimmed mean of M-values (TMM) method^69^. Dispersions were estimated and data were fitted using the edgeR functions estimateDisp and glmQLFit, respectively. Genes were considered as differentially expressed if their Log_2_ fold change expression was significantly (FDR ≤ 0.05) greater than |1|(edgeR function glmTreat). Functional enrichment of significant gene sets was done by hypergeometric testing (R function phyper). Functions were considered as significantly enriched with a FDR adjusted *p*-value ≤ 0.05. The raw sequencing files have been deposited in the NCBI GEO database under the accession number GSE190194.

### Real-time quantitative PCR

Total RNA was extracted using the InviTrap® Spin Cell RNA Mini Kit (INVITE). RNA purity was checked using a NanoDrop spectrophotometer (Thermo Fisher Scientific). cDNA was synthesized by using 1^st^ Strand cDNA Synthesis Kit for RT-PCR (AMV) and RT-qPCR was performed with Sybr green IQ Supermix (Bio-Rad Laboratories), using an iCycler iQ single-color real-time PCR detection system (Bio-Rad Laboratories). Fold changes in expression versus control condition were determined using the 2^(−ΔΔCt)^ method^70^ with *16s-rRNA* as a housekeeping gene (see Supplementary Table 6 for primers sequence).

### Assays of virulence factors in PAO1 supernatants

Elastolytic activity in *P. aeruginosa* culture was determined by elastin Congo red (ECR) assay^71^. Briefly, bacteria from mid-log-phase (OD_620nm_: 0.6–0.8) in MHB-CA were pelleted and re-suspended in fresh MHB-CA in the absence of or presence of potentiators so as to obtain an OD_620nm_ of 0.05, then incubated at 37 °C with rotation of 130 rpm for 21 h. Bacteria were pelleted by centrifugation (20,000 × *g*; 5 min) and the pellet was re-suspended in the original volume of water to measure the OD_620nm_. The supernatants were passed through 0.22 μm filters; 50 μL of filtrates were added to 1 ml of Tris-maleate buffer (0.1 M Tris [pH 7.2], 1 mM CaCl_2_) containing 20 mg of ECR (Sigma). Mixtures were incubated for 18 h at 37 °C with rotation of 130 rpm and then placed on ice after the addition of 0.1 mL of 0.12 M EDTA. Insoluble ECR was removed by centrifugation (20,000 × *g*, 5 min), and the OD_495nm_ was measured. Elastase activity was expressed as (OD_495nm_ of the filtrate/OD_620nm_ of the resuspended pellet), in percentage of the control value (untreated bacteria).

Rhamnolipids were quantified in the culture medium as previously described^71^. Briefly, an aliquot from an overnight culture was added into fresh MHB-CA in the absence of or presence of potentiators to obtain an OD_620 nm_ of 0.05 and incubated at 37 °C with rotation of 130 rpm for 48 h. Then the bacteria were pelleted by centrifugation (20,000 × *g*, 5 min) and the pellet was resuspended in the original volume of water to measure OD_620nm_. Rhamnolipids (containing 3-deoxy-hexose) were assayed in the culture supernatant by the orcinol assay. Rhamnolipids from 300 µL of supernatants were extracted twice with 600 μL diethyl ether (Merck-Millipore). The pooled ether extracts were evaporated to dryness, then reconstituted in 100 μL distilled water and mixed with 100 μL 1.6 % [w/v] orcinol (Sigma) and 800 μL of 60% (v/v) sulfuric acid. After heating to 80 °C and shaking at 175 rpm for 30 min, the OD_421nm_ was measured. The content of rhamnolipids in the samples was expressed as (OD_421nm_ of the supernatant/OD_620nm_ of the resuspended pellet), in percentage of the control value (untreated bacteria).

Pyocyanin was assayed on cultures prepared as for the rhamnolipids assay, but incubated during 10 h only before centrifugation and resuspension of the pellet in water. Pyocyanin was extracted from 5 mL supernatant with 3 mL of chloroform (Merck-Millipore) and then re-extracted into 1 mL of 0.2 N HCl^71^. The absorbance of 520 nm (A_520nm_) was measured and the amount of pyocyanin in the samples was expressed as A_520nm_/OD_620nm_ of the resuspended pellet), in percentage of the control value (untreated bacteria).

Swimming, swarming, and twitching motilities were assessed as previously described^71,72^. Briefly, 2 µL of PAO1 grown in MHB-CA for 14 h were either directly stabbed into swimming (0.1% [w/v] tryptone, 0.05% [w/v] yeast extract (Becton Dickinson), 0.5% [w/v] NaCl, 0.3% [w/v] agar) and twitching (1.0% [w/v] tryptone, 0.5% [w/v] yeast extract, 0.5% [w/v] NaCl, 1.0% [w/v]] agar) plates or diluted in fresh LB medium to obtain an OD_620 nm_ of 0.1 and then spotted onto swarming plates (0.8% [w/v] nutrient broth N.2, 0.5% [w/v] glucose, 0.5% [w/v] agar). Plates were supplemented or not with potentiators. After 24 h of incubation at 37 °C, swimming and swarming motilities were directly observed at the air-agar interface. To better visualize the twitching motility, the plates were immersed with a small volume of cold twitching motility developer solution (TMDS; 400 mL deionized water, 100 mL glacial acetic acid [Merck-Millipore], 500 mL methanol [Merck-Millipore], stored at 4 °C) for 30 min. Then the top colonies were gently scraped with a plastic disposable loop. All images of the plates were taken using a Bio-Rad Molecular imager (Gel Doc XR system).

Biofilm formation was evaluated using cultures grown to mid-log phase in MHB-CA, diluted to 10^6^ CFU/mL in fresh TGN (Trypticase soy broth supplemented with 1% glucose and 2% NaCl), and added by 200 μL bacterial suspension containing or not potentiators was added to 96-well plate. After 24 h of incubation at 37 °C without agitation, the medium was carefully removed and biofilms were washed with 200 μL of 3-morpholinopropane-1-sulfonic acid (MOPS) buffer (20.9 g/liter of MOPS [Sigma-Aldrich], 5.6 g/liter NaCl; pH adjusted to 7 with NaOH). Then the biomass of biofilm was quantified using crystal violet, a cationic dye that non-specifically stains negatively charged constituents in biofilms. Washed biofilms were dried at 60 °C for 30 min and 1 % (v/v) crystal violet (100%, Sigma) was added to the plates. After 10 min of incubation at room temperature, the excess of dye was removed under running water and the residual amount was resolubilized with 66% acetic acid and incubated 1 h at room temperature. Absorbance was then measured at 570 nm using a SpectraMax M3 plate reader and data expressed in percentage of the untreated control.

### Activity against preformed biofilms

Biofilms were formed in 96-well plates^72^. Briefly, overnight cultures of bacteria were diluted in TGN to obtain an OD_620 nm_ of 0.05. Two hundred μL of this suspension were distributed in 96-well plates, which were then incubated at 37 °C during 24 h to obtain mature biofilms. Mature biofilms were then incubated with 200 μL fresh TGN containing rifampicin alone, potentiators alone, or their combination during 24 h, after which the medium was removed and the biofilm was washed once with 200 μL MOPS buffer and biomass quantified by crystal violet staining as described above. The metabolic activity in the biofilms was determined using the fluorescein diacetate (FDA) assay. The non-fluorescent FDA is hydrolyzed by living bacteria into the yellow, highly fluorescent, fluorescein. Washed biofilms were incubated with 100 μg/mL fluorescein diacetate (Sigma-Aldrich) for 15 min at 37 °C in the dark. Fluorescein fluorescence was measured in a SpectraMax M3 plate reader (λexc/λem: 494/518 nm).

### Confocal laser scanning microscopy for visualization of biofilms

Biofilms were grown on round coverslips placed at the bottom of the wells of 24-well plates. After 24 h of incubation, the culture medium was discarded. Biofilms were washed once with 1 mL PBS and reincubated with antibiotics alone or in combination with potentiators for 1 h, washed twice with 1 mL PBS and stained for 30 min in the dark with LIVE/DEAD (SYTO 9/PI) bacterial viability kit (L-7007; Thermo Fisher Scientific) or with 0.5 mM 5-cyano-2,3-ditolyl tetrazolium chloride (CTC, Sigma-Aldrich), as described previously^73^. CTC is a colorless, nonfluorescent, and membrane-permeable compound that is reduced in viable cells to a fluorescent insoluble CTC-formazan. Stained biofilms were then washed with 1 mL PBS to remove the probes in excess.

Biofilms were observed using a cell observation spinning disk microscope (Carl Zeiss) with an oil immersion 40× objective. SYTO9 and NV1532 were detected in the green channel (λexc/λem: 488/502–538 nm), and PI and CTC, in the red channel (λexc/λem: 488/580–654 nm). Images were acquired at a resolution of 1388 × 1040 pixels, and 20-µm-deep scans were obtained using Z-stack scanning mode. 3D-images construction and quantitative analysis of the images were performed using ZEN 2.6 (blue edition) software. For quantification of fluorescent signals, an area of 37,000 µm^2^ was selected on 2D-projection of the image, and the fluorescence intensities of the fluorophores were recorded in all Z-stacks of each sample. The analyses were performed for three different zones of the coverslips, and each experiment was repeated three times.

### Transmission electron microscopy

Samples were prepared as previously described^74^. In brief, PAO1 was incubated in CA-MHB overnight at 37 °C. After centrifugation, bacteria were resuspended in fresh CA-MHB containing NV716 at different concentrations during 1 h. Bacteria were fixed in 1% glutaraldehyde in 0.1 M phosphate buffer for 4 h. After post-fixation in 1% osmium tetroxide for 1 h and dehydration in ethanol, samples were embedded in resin. 70 nm sections were cut with a Leica ultracut UCT ultramicrotome and examined (unstained) in a JEOL 1400 transmission electron microscope equipped with an 11 Mpxl EMSIS Quemesa camera.

### Statistics and reproducibility

Statistical analyses and curve fitting were performed using GraphPad Prism software for Windows (version 9.0.0; GraphPad Software, Inc., San Diego, CA). Most experiments were performed at least in triplicates at 2–4 independent occasions (see details in each figure legend) and data presented as the mean and SEM when applicable. Three samples were prepared for transcriptomic analyses and one sample for genomic or MS analyses. Confocal microscopy analyses were performed on three independent samples with data presented as the mean value; representative images from one sample are shown. Electron microscopy was performed on a single sample but with examination of different zones randomly selected.

### Reporting summary

Further information on research design is available in the Nature Research Reporting Summary linked to this article.

## Supplementary information

Supplementary Material

Description of Additional Supplementary Files

Supplementary Data 1

Supplementary Data 2

Reporting Summary

## Data Availability

The datasets generated during the current study are available as additional file to this paper (Supplementary Data 2). DNAseq and RNAseq data have been deposited in the NCBI SRA database (accession number PRJNA785668) and in the NCBI GEO database (accession number GSE190194), respectively.

## References

[1] Zgurskaya HI, Walker JK, Parks JM, Rybenkov VV (2021). Multidrug efflux pumps and the two-faced janus of substrates and inhibitors. Acc. Chem. Res..

[2] Nikaido H (2003). Molecular basis of bacterial outer membrane permeability revisited. Microbiol Mol. Biol. Rev..

[3] Poole K (2001). Multidrug efflux pumps and antimicrobial resistance in Pseudomonas aeruginosa and related organisms. J. Mol. Microbiol Biotechnol..

[4] Chopra I, Roberts M (2001). Tetracycline antibiotics: mode of action, applications, molecular biology, and epidemiology of bacterial resistance. Microbiol. Mol. Biol. Rev. MMBR.

[5] Vazquez D (1964). Uptake and binding of chloramphenicol by senstitive and resistant organisms. Nature.

[6] MacNair CR, Brown ED (2020). Outer membrane disruption overcomes intrinsic, acquired, and spontaneous antibiotic resistance. mBio.

[7] Lomovskaya O (2001). Identification and characterization of inhibitors of multidrug resistance efflux pumps in Pseudomonas aeruginosa: novel agents for combination therapy. Antimicrob. Agents Chemother..

[8] Morris CM, George A, Wilson WW, Champlin FR (1995). Effect of polymyxin B nonapeptide on daptomycin permeability and cell surface properties in Pseudomonas aeruginosa, Escherichia coli, and Pasteurella multocida. J. Antibiot..

[9] Mahamoud A, Chevalier J, Alibert-Franco S, Kern WV, Pagès JM (2007). Antibiotic efflux pumps in Gram-negative bacteria: the inhibitor response strategy. J. Antimicrob. Chemother..

[10] Danner RL (1989). Purification, toxicity, and antiendotoxin activity of polymyxin B nonapeptide. Antimicrobial agents Chemother..

[11] Douafer H, Andrieu V, Phanstiel OT, Brunel JM (2019). Antibiotic adjuvants: make antibiotics great again!. J. Med. Chem..

[12] Brunel JM, Lieutaud A, Lome V, Pagès JM, Bolla JM (2013). Polyamino geranic derivatives as new chemosensitizers to combat antibiotic resistant gram-negative bacteria. Bioorg. Med. Chem..

[13] Borselli D (2016). Polyamino-isoprenic derivatives block intrinsic resistance of P. aeruginosa to doxycycline and chloramphenicol in vitro. PLoS One.

[14] Borselli D, Brunel JM, Gorgé O, Bolla JM (2019). Polyamino-isoprenyl derivatives as antibiotic adjuvants and motility inhibitors for Bordetella bronchiseptica porcine pulmonary infection treatment. Front. Microbiol..

[15] Wang G, Brunel JM, Bolla JM, Van Bambeke F (2021). The polyaminoisoprenyl potentiator NV716 revives old disused antibiotics against intracellular forms of infection by Pseudomonas aeruginosa. Antimicrob. Agents Chemother..

[16] Lyu Y (2017). Amphiphilic tobramycin-lysine conjugates sensitize multidrug resistant gram-negative bacteria to rifampicin and minocycline. J. Med. Chem..

[17] Yee YC, Kisslinger B, Yu VL, Jin DJ (1996). A mechanism of rifamycin inhibition and resistance in Pseudomonas aeruginosa. J. Antimicrob. Chemother..

[18] Velkov T, Thompson PE, Nation RL, Li J (2010). Structure—activity relationships of polymyxin antibiotics. J. Med. Chem..

[19] Moore RA, Bates NC, Hancock RE (1986). Interaction of polycationic antibiotics with Pseudomonas aeruginosa lipopolysaccharide and lipid A studied by using dansyl-polymyxin. Antimicrobial Agents Chemother..

[20] Zorko M, Jerala R (2008). Alexidine and chlorhexidine bind to lipopolysaccharide and lipoteichoic acid and prevent cell activation by antibiotics. J. Antimicrob. Chemother..

[21] Jagtap P (2018). Mechanistic evaluation of lipopolysaccharide-alexidine interaction using spectroscopic and in silico approaches. ACS Infect. Dis..

[22] Chawner JA, Gilbert P (1989). Interaction of the bisbiguanides chlorhexidine and alexidine with phospholipid vesicles: evidence for separate modes of action. J. Appl Bacteriol..

[23] Olaitan AO, Morand S, Rolain JM (2014). Mechanisms of polymyxin resistance: acquired and intrinsic resistance in bacteria. Front Microbiol.

[24] Loh B, Grant C, Hancock RE (1984). Use of the fluorescent probe 1-N-phenylnaphthylamine to study the interactions of aminoglycoside antibiotics with the outer membrane of Pseudomonas aeruginosa. Antimicrob. Agents Chemother..

[25] Sautrey G (2014). New amphiphilic neamine derivatives active against resistant Pseudomonas aeruginosa and their interactions with lipopolysaccharides. Antimicrob. Agents Chemother..

[26] Brazas MD, Hancock RE (2005). Ciprofloxacin induction of a susceptibility determinant in Pseudomonas aeruginosa. Antimicrob. Agents Chemother..

[27] Hudson MA, Lockless SW (2022). Elucidating the mechanisms of action of antimicrobial agents. mBio.

[28] Moradali MF, Ghods S, Rehm BH (2017). Pseudomonas aeruginosa lifestyle: a paradigm for adaptation, survival, and persistence. Front Cell Infect. Microbiol.

[29] Donlan RM (2002). Biofilms: microbial life on surfaces. Emerg. Infect. Dis..

[30] Tsubery H, Ofek I, Cohen S, Eisenstein M, Fridkin M (2002). Modulation of the hydrophobic domain of polymyxin B nonapeptide: effect on outer-membrane permeabilization and lipopolysaccharide neutralization. Mol. Pharm..

[31] Khadka NK, Aryal CM, Pan J (2018). Lipopolysaccharide-dependent membrane permeation and lipid clustering caused by cyclic lipopeptide colistin. ACS Omega.

[32] Jiang X (2020). Molecular dynamics simulations informed by membrane lipidomics reveal the structure-interaction relationship of polymyxins with the lipid A-based outer membrane of Acinetobacter baumannii. J. Antimicrob. Chemother..

[33] Dupuy FG (2018). Selective interaction of colistin with lipid model membranes. Biophys. J..

[34] Pajouhesh H, Lenz GR (2005). Medicinal chemical properties of successful central nervous system drugs. NeuroRx.

[35] Fernandez L (2013). Characterization of the polymyxin B resistome of Pseudomonas aeruginosa. Antimicrob. Agents Chemother..

[36] Chadha J, Harjai K, Chhibber S (2022). Revisiting the virulence hallmarks of Pseudomonas aeruginosa: a chronicle through the perspective of quorum sensing. Environ. Microbiol..

[37] Chadha, J., Harjai, K. & Chhibber, S. Revisiting the virulence hallmarks of Pseudomonas aeruginosa: a chronicle through the perspective of quorum sensing. *Environ. Microbiol*. **24**, 2630–2656 (2022). 10.1111/1462-2920.15784 34559444

[38] Chen L, Zou Y, Kronfl AA, Wu Y (2020). Type VI secretion system of Pseudomonas aeruginosa is associated with biofilm formation but not environmental adaptation. Microbiologyopen.

[39] Yasir M, Dutta D, Willcox MDP (2019). Comparative mode of action of the antimicrobial peptide melimine and its derivative Mel4 against Pseudomonas aeruginosa. Sci. Rep..

[40] Klubthawee N, Adisakwattana P, Hanpithakpong W, Somsri S, Aunpad R (2020). A novel, rationally designed, hybrid antimicrobial peptide, inspired by cathelicidin and aurein, exhibits membrane-active mechanisms against Pseudomonas aeruginosa. Sci. Rep..

[41] Elliott AG (2020). An amphipathic peptide with antibiotic activity against multidrug-resistant Gram-negative bacteria. Nat. Commun..

[42] Aoki W, Ueda M (2013). Characterization of antimicrobial peptides toward the development of novel antibiotics. Pharmaceuticals.

[43] Lei J (2019). The antimicrobial peptides and their potential clinical applications. Am. J. Transl. Res..

[44] Abdi M, Mirkalantari S, Amirmozafari N (2019). Bacterial resistance to antimicrobial peptides. J. Pept. Sci..

[45] Zimmermann L (2018). Broad-spectrum antibacterial amphiphilic aminoglycosides: a new focus on the structure of the lipophilic groups extends the series of active dialkyl neamines. Eur. J. Med. Chem..

[46] Defraine V (2018). 1-((2,4-Dichlorophenethyl)Amino)-3-Phenoxypropan-2-ol kills pseudomonas aeruginosa through extensive membrane damage. Front. Microbiol..

[47] Fontanay S, Grare M, Mayer J, Finance C, Duval RE (2008). Ursolic, oleanolic and betulinic acids: antibacterial spectra and selectivity indexes. J. Ethnopharmacol..

[48] Stover CK (2000). Complete genome sequence of Pseudomonas aeruginosa PAO1, an opportunistic pathogen. Nature.

[49] Köhler T, Michea-Hamzehpour M, Plesiat P, Kahr AL, Pechere JC (1997). Differential selection of multidrug efflux systems by quinolones in Pseudomonas aeruginosa. Antimicrob. Agents Chemother..

[50] Mima T, Joshi S, Gomez-Escalada M, Schweizer HP (2007). Identification and characterization of TriABC-OpmH, a triclosan efflux pump of Pseudomonas aeruginosa requiring two membrane fusion proteins. J. Bacteriol..

[51] Jacobs MA (2003). Comprehensive transposon mutant library of Pseudomonas aeruginosa. Proc. Natl Acad. Sci. USA.

[52] Lieutaud A, Pieri C, Bolla JM, Brunel JM (2020). New polyaminoisoprenyl antibiotics enhancers against two multidrug-resistant gram-negative bacteria from enterobacter and salmonella species. J. Med. Chem..

[53] Odds FC (2003). Synergy, antagonism, and what the chequerboard puts between them. J. Antimicrob. Chemother..

[54] Swain J (2019). Antimicrobial activity of amphiphilic neamine derivatives: understanding the mechanism of action on Gram-positive bacteria. Biochim Biophys. Acta Biomembr..

[55] Ocaktan A, Yoneyama H, Nakae T (1997). Use of fluorescence probes to monitor function of the subunit proteins of the MexA-MexB-oprM drug extrusion machinery in Pseudomonas aeruginosa. J. Biol. Chem..

[56] Sautrey G (2016). Negatively charged lipids as a potential target for new amphiphilic aminoglycoside antibiotics: a biophysical study. J. Biol. Chem..

[57] Chapman JS, Georgopapadakou NH (1989). Fluorometric assay for fleroxacin uptake by bacterial cells. Antimicrob. Agents Chemother..

[58] Fenosa A (2009). Role of TolC in Klebsiella oxytoca resistance to antibiotics. J. Antimicrob. Chemother..

[59] Drugeon HB, Juvin ME, Bryskier A (1999). Relative potential for selection of fluoroquinolone-resistant Streptococcus pneumoniae strains by levofloxacin: comparison with ciprofloxacin, sparfloxacin and ofloxacin. J. Antimicrob. Chemother..

[60] Andrews, S. A quality control tool for high throughput sequence data, http://www.bioinformatics.babraham.ac.uk/projects/fastqc/ (2010).

[61] Treangen TJ, Ondov BD, Koren S, Phillippy AM (2014). The Harvest suite for rapid core-genome alignment and visualization of thousands of intraspecific microbial genomes. Genome Biol..

[62] Shishkin AA (2015). Simultaneous generation of many RNA-seq libraries in a single reaction. Nat. Methods.

[63] Bhattacharyya RP (2019). Simultaneous detection of genotype and phenotype enables rapid and accurate antibiotic susceptibility determination. Nat. Med..

[64] Martin M (2011). Cutadapt removes adapter sequences from high-throughput sequencing reads. EMBnet. J..

[65] Langmead B, Salzberg SL (2012). Fast gapped-read alignment with Bowtie 2. Nat. Methods.

[66] Li H (2009). The sequence alignment/map format and SAMtools. Bioinformatics.

[67] Liao Y, Smyth GK, Shi W (2014). featureCounts: an efficient general purpose program for assigning sequence reads to genomic features. Bioinformatics.

[68] Robinson MD, McCarthy DJ, Smyth GK (2010). edgeR: a bioconductor package for differential expression analysis of digital gene expression data. Bioinformatics.

[69] Robinson MD, Oshlack A (2010). A scaling normalization method for differential expression analysis of RNA-seq data. Genome Biol..

[70] Livak KJ, Schmittgen TD (2001). Analysis of relative gene expression data using real-time quantitative PCR and the 2(-Delta Delta C(T)) method. Methods.

[71] Imperi F (2013). New life for an old drug: the anthelmintic drug niclosamide inhibits Pseudomonas aeruginosa quorum sensing. Antimicrob. Agents Chemother..

[72] Diaz Iglesias Y, Van Bambeke F (2020). Activity of antibiotics against pseudomonas aeruginosa in an in vitro model of biofilms in the context of cystic fibrosis: influence of the culture medium. Antimicrob. Agents Chemother..

[73] Siala W (2014). Comparison of the antibiotic activities of Daptomycin, Vancomycin, and the investigational Fluoroquinolone Delafloxacin against biofilms from Staphylococcus aureus clinical isolates. Antimicrob. Agents Chemother..

[74] Alhanout K (2010). New insights into the antibacterial mechanism of action of squalamine. J. Antimicrob. Chemother..

[75] Schulz M, Iwersen-Bergmann S, Andresen H, Schmoldt A (2012). Therapeutic and toxic blood concentrations of nearly 1000 drugs and other xenobiotics. Crit. Care.

[76] Ruslami R (2007). Pharmacokinetics and tolerability of a higher rifampin dose versus the standard dose in pulmonary tuberculosis patients. Antimicrob. Agents Chemother..

