# Supplementary material for: The membrane-active polyaminoisoprenyl compound NV716 re-sensitizes Pseudomonas aeruginosa to antibiotics and reduces bacterial virulence
Source: Commun Biol. 2022 Aug 25;5:871. doi: 10.1038/s42003-022-03836-5 (PMC9411590; doi:10.1038/s42003-022-03836-5)
Supplement: Supplementary file 1 — Supplementary Material [file 42003_2022_3836_MOESM1_ESM.docx]

**The membrane-active polyaminoisoprenyl compound NV716 re-sensitizes *Pseudomonas aeruginosa* to antibiotics and reduces bacterial virulence**

Gang Wang^1^, Jean-Michel Brunel^2^, Matthias Preusse^3^, Negar Mozaheb^1^, Sven D. Willger^3,4^, Gerald Larrouy-Maumus^5^, Pieter Baatsen^6^, Susanne Häussler^3,4,7,8^, Jean-Michel Bolla^2^, Françoise Van Bambeke^1^

^1^ Pharmacologie cellulaire et moléculaire, Louvain Drug Research Institute, Université catholique de Louvain, Brussels, Belgium

^2^ Aix Marseille Université, INSERM, SSA, *Membranes et Cibles thérapeutiques* (MCT), Marseille, France.

^3^ Department of Molecular Bacteriology, Helmoltz Centre for Infection Research, Braunschweig, Germany
^4^ Department of Molecular Bacteriology, Twincore, Hannover, Germany

^5^ Department of Life Sciences, Faculty of Natural Sciences, MRC Centre for Molecular Bacteriology and Infection, Imperial College London, London, United Kingdom

^6^ Electron Microscopy Platform & Bio Imaging Core, VIB & KULeuven Center for Brain & Disease Research, KULeuven, Belgium

^7^ Department of Clinical Microbiology, Rigshospitalet, Copenhagen, Denmark

^8^ Cluster of Excellence RESIST, Hannover Medical School, Hannover, Germany

**Supplementary material**

**Supplementary Table 1: MICs of potentiators against the strains used in Figure 3**.

| Strain identification | MIC (µM /[mg/L]) | | | | | |
| --- | --- | --- | --- | --- | --- | --- |
|  | ALE | PMBN | CST | PAβN | NV731 | NV716 |
| PAO1 | 12 / 7 | 128 / 128 | 1 / 1 | >250 / >125 | >250 / >80 | 50/ 21 |
| PAO509 | 6 / 3.5 | 128 / 128 | 1 / 1 | >250 / >125 | >250 / >80 | 25 / 10.5 |
| 2938 | 12 / 7 | >256 / >256 | 64 / 64 | >250 / >125 | >250 / >80 | 50 / 21 |
| 307 | 12 / 7 | >256 / >256 | 64 / 64 | >250 / >125 | >250 / >80 | 50 / 21 |

**Supplementary Table 2** : **SNPs identified in PAO1 strains** after 52 passages in the presence of potentiators at ¼ MIC compared to the untreated control.

| **PAO1 control** | **NV716** | **Colistin** | **Alexidine** | **Genome location (gene, intergenic)** | **Synonymous (S) / Nonsynonymous (N)** |
| --- | --- | --- | --- | --- | --- |
| 0 | 1 | 1 | 1 | intergenic between PA5015 and PA5016 | N/A |
| 0 | 0 | 1 | 1 | PA5267 (Hcp) | S |
| 0 | 0 | 1 | 1 | PA5267 (Hcp) | S |
| 0 | 0 | 1 | 0 | PA1180 (PhoQ) | N (V260G) |
| 0 | 0 | 0 | 1 | intergenic between PA3811 and PA3810 | N/A |
| 0 | 0 | 1 | 0 | PA1561 (Aer) | N (G428D) |
| 0 | 0 | 0 | 1 | intergenic between PA1843 and PA1844 (Tse) | N/A |
| 0 | 1 | 0 | 0 | intergenic between PA1843 and PA1844 (Tse) | N/A |
| 0 | 1 | 0 | 0 | PA4601 (MorA) | N (A1149V) |
| 0 | 0 | 0 | 1 | PA4493 (RoxR) | N (D116E) |
| 0 | 1 | 0 | 0 | PA4493 (RoxR) | N (A123V) |
| 0 | 0 | 1 | 0 | PA1511 (VgrG2a) | S |
| 0 | 1 | 1 | 1 | intergenic between PA2348 and PA2327 | N/A |
| 0 | 0 | 1 | 0 | PA4777 (PmrB) | N (M48I) |
| 0 | 0 | 0 | 1 | PA4270 (RpoB) | N (Q468P) |

**Supplementary Table 3** : **DEGs involved in elastase, rhamnolipid and pyocyanin production in PAO1** after 52 passages in the presence of potentiators at ¼ MIC. Down-regulated genes are highlighted in blue; upregulated genes, in red. Genes related to QS are in bold.

| Regulated elements | LOCUS TAG | Name | NV716 | | Colistin | | Alexidine | |
| --- | --- | --- | --- | --- | --- | --- | --- | --- |
|  |  |  | Log_2_FC^a^ | FDR^b^ | Log_2_FC | FDR | Log_2_FC | FDR |
| Elastase | **PA3724** | ***lasB*** | -2.87 | 3.40E-15 | 1.25 | 0.01656 | -2.50 | 1.11E-15 |
|  | **PA1871** | ***lasA*** | -1.76 | 1.10E-05 | 1.28 | 0.01215 | -1.84 | 2.38E-08 |
|  | **PA1432** | ***lasI*** | -1.99 | 1.80E-12 |  |  | -1.36 | 5.83E-05 |
| Rhamnolipid | **PA3479** | ***rhlA*** | -2.97 | 5.30E-13 |  |  |  |  |
|  | **PA3478** | ***rhlB*** | -3.69 | 7.30E-22 |  |  | -2.13 | 7.30E-14 |
|  | **PA1130** | ***rhlC*** | -1.38 | 0.03808 |  |  |  |  |
|  | **PA3476** | ***rhlI*** | -1.66 | 0.00106 | 2.08 | 1.90E-11 |  |  |
|  | **PA0996** | ***pqsA*** | -5.46 | 6.72E-32 |  |  |  |  |
|  | **PA0997** | ***pqsB*** | -4.57 | 2.70E-26 |  |  | -1.43 | 2.90E-05 |
|  | **PA0998** | ***pqsC*** | -4.11 | 7.10E-21 |  |  | -1.30 | 0.00871 |
|  | **PA0999** | ***pqsD*** | -3.52 | 4.28E-25 |  |  | -1.45 | 2.70E-06 |
|  | **PA1000** | ***pqsE*** | -4.68 | 2.18E-33 |  |  | -2.28 | 1.00E-21 |
|  | **PA1001** | ***phnA*** | -4.88 | 6.91E-07 |  |  | -2.09 | 0.00011 |
|  | **PA1002** | ***phnB*** | -3.18 | 1.54E-18 |  |  | -2.29 | 4.11E-15 |
|  | **PA2587** | ***pqsH*** | -1.68 | 0.00116 |  |  |  |  |
|  | **PA1431** | ***rsaL*** | -2.43 | 2.53E-15 | 1.30 | 0.00093 | -1.80 | 6.15E-11 |
|  | PA2656 | *bqsS* | 2.85 | 1.58E-22 |  |  | 1.26 | 0.00799 |
|  | PA2657 | *bqsR* | 4.64 | 4.07E-37 | 1.42 | 1.63E-05 | 2.33 | 3.74E-20 |
|  | PA5360 | *phoB* | 1.97 | 1.94E-07 |  |  | 1.70 | 8.74E-06 |
|  | **PA2591** | ***vqsR*** | -2.09 | 9.84E-07 |  |  |  |  |
|  | PA1898 | *qscR* |  |  | -3.02 | 1.77E-20 | -2.11 | 4.40E-12 |
|  | PA2586 | *gacA* |  |  | -1.39 | 2.02E-07 |  |  |
|  | PA4723 | *dksA* |  |  |  |  | 1.33 | 0.00517 |
| Pyocyanin | **PA1905** | ***phzG2*** | -5.14 | 8.40E-10 |  |  | -5.15 | 2.70E-13 |
|  | **PA4210** | ***phzA1*** | -6.31 | 0.0324 |  |  |  |  |
|  | **PA4211** | ***phzB1*** |  |  | 4.31 | 1.37E-11 |  |  |
|  | **PA4216** | ***phzG1*** | -4.75 | 1.60E-10 |  |  | -3.32 | 1.80E-10 |
|  | **PA4217** | ***phzS*** | -5.55 | 8.90E-35 |  |  | -3.66 | 2.20E-32 |
| Type VI protein secretion system | **PA1657** | ***HsiB2*** | -3.32 | 6.31739E-13 | 2.74 | 4.6E-19 |  |  |
|  | **PA1658** | ***HsiC2*** | -2.53 | 1.96916E-12 | 2.29 | 3.21E-17 |  |  |
|  | **PA1659** | ***HsiF2*** | -3.30 | 3.20907E-10 | 2.27 | 1.82E-11 |  |  |
|  | **PA1660** | ***HsiG2*** | -3.51 | 3.92903E-22 |  |  | -2.09 | 1.495E-14 |
|  | **PA1661** | ***HsiH2*** | -3.44 | 2.51619E-21 |  |  | -2.69 | 3.136E-20 |
|  | **PA1662** | ***clpv2*** | -2.67 | 1.25029E-14 |  |  | -1.69 | 2.918E-08 |
|  | **PA1663** | ***sfa2*** | -3.05 | 2.42559E-17 |  |  | -2.16 | 1.8E-13 |
|  | **PA1665** | ***HsiH2*** | -3.32 | 7.73637E-20 |  |  | -2.28 | 1.291E-15 |
|  | **PA1666** | ***lip2*** | -4.05 | 2.14885E-14 |  |  | -1.84 | 5.806E-06 |
|  | **PA1668** | ***DotU2*** | -2.33 | 5.48318E-11 |  |  | -2.35 | 4.63E-14 |
|  | **PA1669** | ***IcmF2*** | -2.35 | 1.01006E-17 |  |  | -1.66 | 1.129E-10 |
|  | **PA1670** | ***stp1*** | -2.99 | 2.84877E-17 |  |  | -2.18 | 8.459E-14 |
|  | PA1671 | *stk1* | -3.19 | 4.70683E-15 |  |  | -2.30 | 2.102E-12 |

^a^ logarithm of fold change ratio
^b^ false discovery rate

**Supplementary Table 4:**  **MICs/MBCs of antibiotics against PAO1 and its deletion mutants in the absence or in the presence of 2.5 µM NV716.** MICs/MBCs reduced of at least 2 dilutions vs. PAO1 are shown in red.

| Strains (inactivated process or synthesis pathway) | Gene deleted | MIC/MBC | | | | | | | |
| --- | --- | --- | --- | --- | --- | --- | --- | --- | --- |
|  |  | DOX | | CHL | | RIF | | CIP | |
|  |  | ABs alone | +NV716 (2.5µM) | ABs alone | +NV716 (2.5µM) | ABs alone | +NV716 (2.5µM) | ABs alone | +NV716 (2.5µM) |
| PAO1 (WT) |  | 16/96 | 2/8 | 32/192 | 2/16 | 16/80 | 0.25/2 | 0.25/0.75 | 0.06/0.25 |
| PW7020 (lipid A) | *arnB* | 32/64 | 0.25/1 | 32/160 | 1/4 | 16/32 | 0.03/0.13 | 0.25/0.25 | 0.06/0.25 |
| PW7302 (Elastase) | *lasB* | 32/64 | 1/4 | 32/160 | 2/8 | 16/64 | 0.25/1 | 0.13/0.25 | 0.13/0.25 |
| PW3959 (T6SS) | *clpV2* | 32/64 | 1/4 | 32/160 | 2/8 | 16/64 | 0.25/1 | 0.13/0.25 | 0.13/0.25 |
| PW6886 (Rhamnolipid) | *rhlA* | 32/64 | 1/2 | 32/160 | 2/8 | 16/32 | 0.25/0.5 | 0.13/0.25 | 0.06/0.13 |
| PW2806 (Pyocyanin) | *pqsE* | 32/64 | 1/4 | 32/160 | 2/8 | 16/80 | 0.25/1 | 0.13/0.25 | 0.13/0.25 |
| PW6880 (Biofilm formation) | *rhlI* | 32/64 | 1/4 | 32/160 | 1/4 | 16/32 | 0.25/1 | 0.13/0.25 | 0.13/0.25 |
| PW2813 (Biofilm formation) | *mvfR* | 32/64 | 1/4 | 32/160 | 2/8 | 16/32 | 0.25/1 | 0.13/0.25 | 0.13/0.25 |
| PW3601 | *lasI* | 32/64 | 1/4 | 32/160 | 2/8 | 16/32 | 0.25/1 | 0.13/0.25 | 0.13/0.25 |
| PW6883 | *rhlR* | 32/64 | 1/4 | 32/160 | 2/8 | 16/64 | 0.25/1 | 0.13/0.25 | 0.13/0.25 |
| PW3597 | *lasR* | 32/64 | 1/4 | 32/160 | 2/8 | 16/32 | 0.25/1 | 0.13/0.25 | 0.13/0.25 |
| PW9024 | *pmrB* | 32/64 | 0.5/2 | 32/160 | 2/8 | 16/32 | 0.25/1 | 0.13/0.25 | 0.13/0.25 |

None of the deletion mutants showed a significant difference in susceptibility to antibiotics alone or combined with NV716, with the exception of doxycycline and rifampicin in the *arnB* mutant and doxycycline in the *pmrB* mutant). MBCs were in general slightly lower in the mutants than in PAO1.

**Supplementary Table 5. comparison of structural properties of the potentiators used in this study** (estimated with MarvinSketch 20.6, Chemaxon)

| **Parameters** | **Alexidine** | **PMBN** | **Colistin** | **NV716** | **NV731** | **PAβN** |
| --- | --- | --- | --- | --- | --- | --- |
| **LogD (pH 7.4)** | -3.9 | -19.1 | -20.2 | -4.2 | -6.3 | -1.2 |
| **Van der Waals molecular Surface Area (Å^2^)** | 932.33 | 1450.38 | 1835.51 | 794.91 | 633.42 | 655.21 |
| **Polar Surface Area (Å^2^)** | 167.58 | 432.46 | 490.66 | 62.11 | 67.31 | 146.12 |
| **Van der Waals volume (Å^3^)** | 543.41 | 901.17 | 1113.8 | 462.27 | 368.73 | 415.59 |

**Supplementary Table 6** : **Primers for quantitative real-time PCR**

| Gene numbering | Gene name | Primer sequences (5‘ to 3’)  (The primers were designed using the Primer3 program (*https://primer3.ut.ee/*)) |
| --- | --- | --- |
| PA1000 | *pqsE* | Forward: TGATGACCTGTGCCTGTTGG |
|  |  | Reverse: GGCTGATCCCTCCTTCAACC |
| PA3479 | *rhlA* | Forward: CCTGGCCGAACATTTCAACG |
|  |  | Reverse: TTTCCACCTCGTCGTCCTTG |
| PA1662 | *clpV2* | Forward: AGATCTTCGACAAGGGCGTG |
|  |  | Reverse: CGAGGTTGGAGGTCATCAGG |
| PA3724 | *lasB* | Forward: TGAACGACGCGCATTTCTTC |
|  |  | Reverse: CCCGTAGTGCACCTTCATGT |
| [PA3617](https://www.pseudomonas.com/feature/show?id=110053) | *recA* | Forward: AGGCCGAGTTCCAGATCCT |
|  |  | Reverse: CTTCTCGACCAGGCCCAATT |
| PA4280.5 | *16S rRNA* | Forward: TACCTGGCCTTGACATGCTG |
|  |  | Reverse: CCCAACATCTCACGACACGA |
| PA3552 | *arnB* | Forward: ACTTTCTGCCATTCTCCCGG |
|  |  | Reverse: TTCGAGCTCCTGGTTCTTCG |

**Supplementary Figure 1: Chemical structures of potentiators** **used in this study.** The aminated functions of potentiators that are partially protonated at physiological pH are evidenced by blue rectangles.

**Supplementary** **Figure 2: LPS modifications in colistin-resistant isolates.** Representative mass spectra of susceptible and modified *Pseudomonas aeruginosa* lipid A acquired using the linear negative-ion mode of a matrix-assisted laser desorption ionization (MALDI) Biotyper Sirius system (Bruker Daltonics). Susceptible *P. aeruginosa* PAO1 lipid A is detected as two major peaks at *m/z* 1,446.7 and *m/z* 1,462.7. Lipid A from colistin-resistant isolate PA2938 with additional peak at *m/z* 1577.9 (additional 4-amino-L-arabinose (L-Ara4N)), isolate PA313, with additional peaks at *m/z* 1657.1 (additional C14:0) and *m/z* 1788.3 (additional L-Ara4N), isolates PA272 and PA307 with additional peaks at *m/z* 1617 (additional 3-OH C10:0) and *m/z* 1748.1 (additional L-Ara4N). L-Ara4N residues are shown in red and additional Carbon chains in blue. The mutations in these strains have been published earlier^1^ and are reproduced in the table below the figure, together with the changes in lipid A observed here.

**
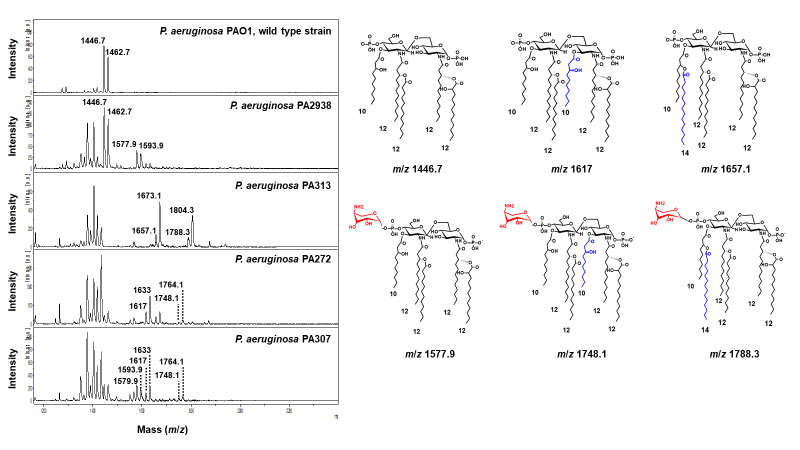
**

|  | **Mutations (vs PAO1)** | | | | **Changes in lipid A (vs PAO1)** |
| --- | --- | --- | --- | --- | --- |
| **Isolates** | *phoQ* | *pmrB* | *parS* | *parR* |  |
| PA2938 | C68T (Ala23Val) | G139A (Asp47Asn) T1033C (Tyr345His) | G1245A (His398Arg) | - | L-Ara4N |
| PA272 | C68T (Ala23Val) | T83C (Val28Ala) T1033C (Tyr345His) | - | - | C14:0 (C3’ position) +/- L-Ara4N |
| PA307 | C68T (Ala23Val) | T83C (Val28Ala) T1033C (Tyr345His) | - | - | 3-OH-C10:0 (C3 position) +/- L-Ara4N |
| PA313 | C68T (Ala23Val) | T485C (Leu162Pro) T1033C (Tyr345His) | - | - | 3-OH-C10:0 (C3 position) +/- L-Ara4N |

Method for identification of LPS modifications in colistin-resistant isolates. Modifications in lipid A were identified by MALDIxin test, exactly as previously described^2^, except that the acidic hydrolysis step was prolonged during 2 h because of the mucoid character of some isolates.

**Supplementary Figure 3. Influence of Mg^2+^ concentration on the binding of potentiators to LPS**. **a-e.** LPS in solution; **f-k.** whole bacteria (PAO1). Potentiators were used at a fixed concentration (10 μM for alexidine, colistin, and NV716; 30 μM for PMBN, 50 μM for PAβN and 25 μM for NV731). The dotted line shows the value for the corresponding control (without Mg^2+^). All data are mean ± SEM (triplicates from 3 independent experiments). Statistical analysis: one-way ANOVA with Dunnett’s post-hoc test (NS: no significant [p>0.05]; *, p <0.05; **, P<0.01; *** p<0.001; **** p<0.0001).

Method for assay of binding to LPS in solution : stock solutions of 500 μM BC and 5 mg/mL LPS extracted from *Pseudomonas aeruginosa* serotype 10 (Sigma-Aldrich) were prepared in Tris buffer (50 mM, pH 7.4), then mixed to reach the final desired concentrations (BC: 5 μM; LPS: 10 μg/mL) and maintained in the dark at room temperature. After 4 hours of incubation, 50 μL of a solution of the test compounds were added to 50 µL of this mix in a 96-wells black plate, which was kept for 30 mins in the dark at room temperature. Fluorescence was read (λexc/λem: 580/620 nm) in a SpectraMax M3 plate reader (Molecular Devices LLC, Sunnyvale, CA).

**Supplementary Figure 4. Effect of alexidine, colistin, and NV716 on inner membrane permeability**. Each compound was used at a concentration of 1 x MIC (See Supplementary Table 1). Inner membrane permeability as assessed by measuring the fluorescence of propidium iodide (PI) after 1 h of incubation with PAO1, PAO509, or two colistin-resistant clinical isolates (PA307 and PA2938). The effect measured with 0.5 % SDS (w/v) after 1 h was taken as 100% (positive control). All data are means ± SEM (triplicates from 3 independent experiments). Statistical analysis: two-way ANOVA with Tukey post-hoc test: ****, p<0.0001; ***, p< 0.001; **: p<0.01.

**Supplementary Figure 5. Transmission electron microscopy images of PAO1**. **a.** control conditions; **b.** after 1 h of incubation with NV716 at 10 µM. Bars: 1 µm (left) or 200 nm (right).

| **a**  **b** |  |
| --- | --- |

**Supplementary Figure 6. Chemical structure of the fluorescent derivative of rifampicin used in this study** (NV1532).

**Supplementary Figure 7**. **Volcano plot of the differentially expressed genes (DEGs) in PAO1 after serial passages**. Bacteria were cultivated over 52 passages in the presence of NV716 (**a**), colistin (CST; **b**) or alexidine (ALE; **c**) at ½ MIC. The x- and y-axes represent the level of change in expression (log_2_ Fold Change scale) and the degree of statistical significance (negative log_10_ transformed False Discovery Rate), respectively. Red dots represent genes which are significantly regulated (FDR ≤ 0.05; horizontal dotted line) to levels at least 2-fold (vertical dotted line) higher or lower than the untreated control (function glmTreat of the R package edgeR).

**Supplementary Figure 8. Outer membrane permeability of PAO1 and of its deletion mutants** (see Supplementary Table 4). The rate of 1-*N*-phenylnaphthylamine (NPN) uptake was measured at early-time points (0-4 seconds). All data are the mean ± SEM (triplicates from three independent experiments). Statistical analysis: one-way ANOVA with Dunnett’s post-hoc test for comparison between PAO1 and each mutant: no significant difference was observed.

No significant difference was observed in the outer membrane permeability of the mutants vs. PAO1 as assessed by measuring the NPN uptake (same conditions as in Figure 3).

**Supplementary Figure 9.** **a-b. Influence of potentiators on metabolic activity.** Fluorescein diacetate metabolization was evaluated in planktonic cultures of PAO1 (**a**) and PAO509 (**b**), in the same conditions as those described for the biofilm assay (Figure 5, incubation during 15 min with potentiators alone, rifampicin alone at 1 x or 5 x MIC, and their combination). **c-d.** CFUs counts in the same conditions. All data are mean ± SEM (triplicates from three independent experiments). Statistical analysis: two-way ANOVA with Tukey post-hoc test: ****, p<0.0001; ***, p< 0.001; **: p<0.01.

An increase in fluorescence signal is observed for agents causing a permeabilization of the outer membrane, suggesting it is related to an increased uptake of the dye inside bacteria. These increase in fluorescence occurs without increase in bacterial counts (rather a decrease when bacteria are exposed to rifampicin at 5 x MIC alone or in combination).

**Supplementary Figure 10.**  **Influence of NV716 on persisters selected by ciprofloxacin (a-b**) **or rifampicin** (**c-d**). Kinetics of killing of stationary phase cultures of PAO1 by ciprofloxacin or rifampicin at 50xMIC alone or combined with NV716 at 10 µM. NV716 was added at different timings (0h [**a,c**], or 5h [**b,d**]), highlighted by the black arrow. All data are expressed as means ± SEM (triplicates from 3 experiments). Statistical analysis: Student's t-test: ** P ≤ 0.01; ***, P ≤ 0.001.

**Supplementary Figure 11:** **Three-dimensional structure of the potentiators PAβN, NV731, and NV716**. Each panel shows the Van der Waals 2 D surface and the ball-and-stick representation (drawn with Hyperchem Pro 6, Hypercube Inc. ).

| **PAβN** | |
| --- | --- |
| 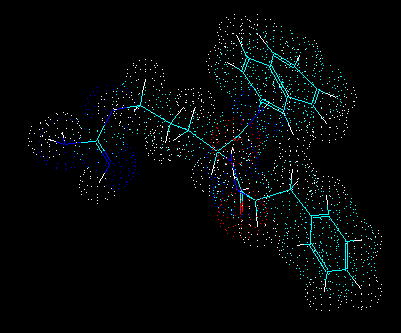 | 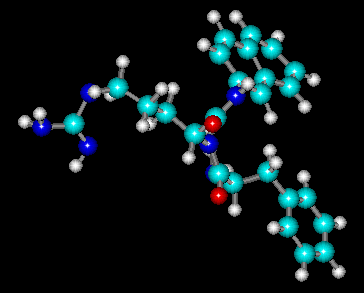 |
| **NV731** | |
| 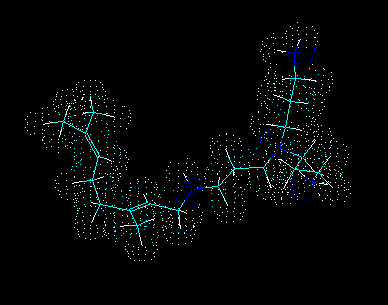 | 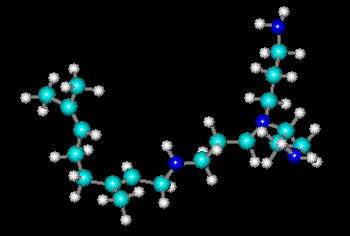 |
| **NV716** | |
|  |  |
| 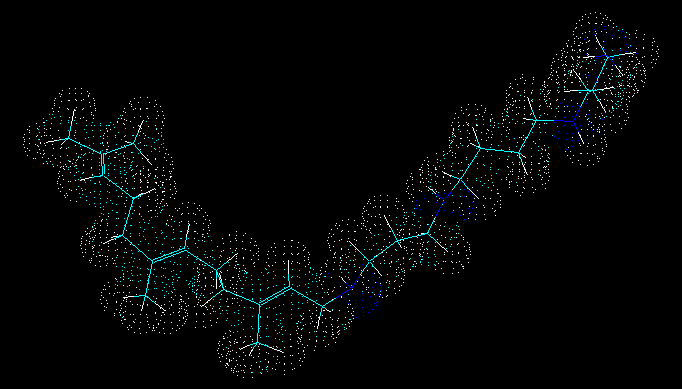 | 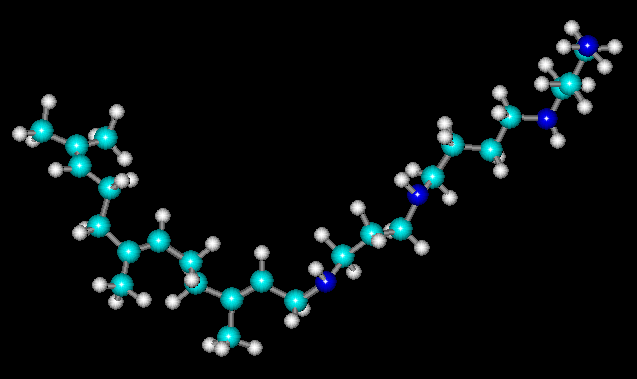 |

**Supplementary Figure 12: Three-dimensional structure of the potentiators PMBN, colistin, and alexidine**. Each panel shows the Van der Waals 2 D surface and the ball-and-stick representation (drawn with Hyperchem Pro 6, Hypercube Inc. )

| **PMBN** | |
| --- | --- |
| **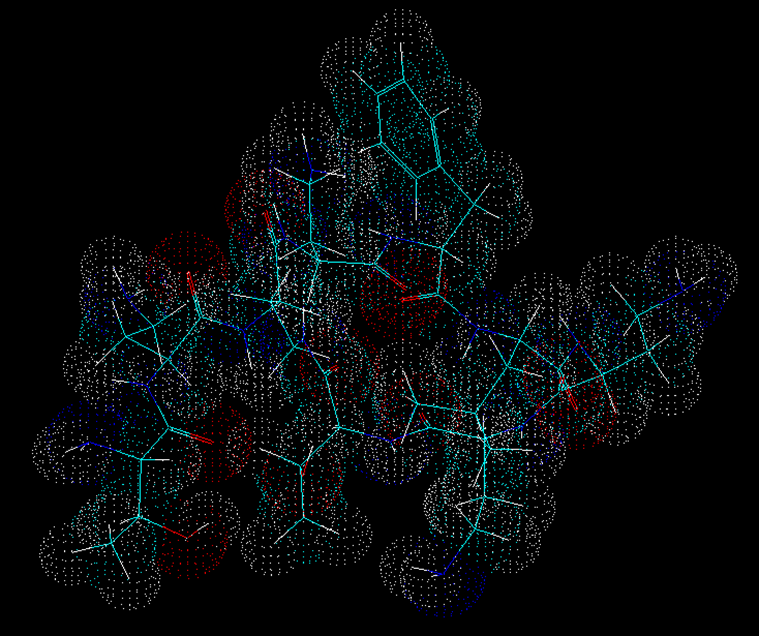** | 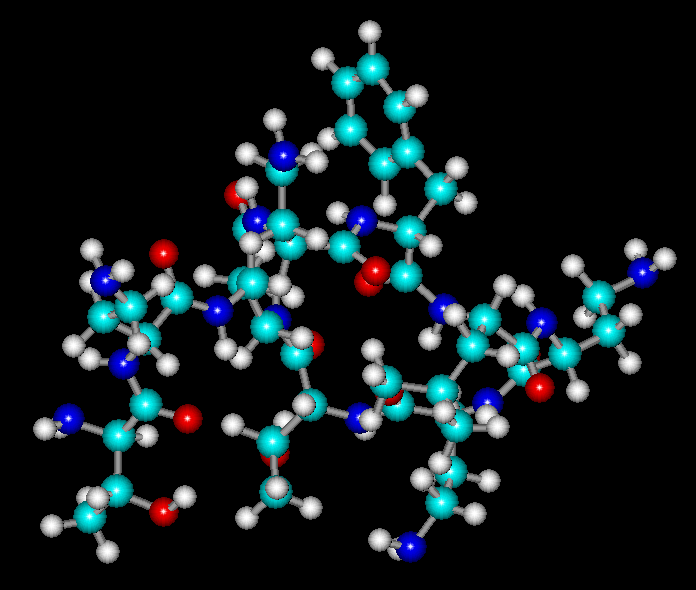 |
| **colistin** | |
| 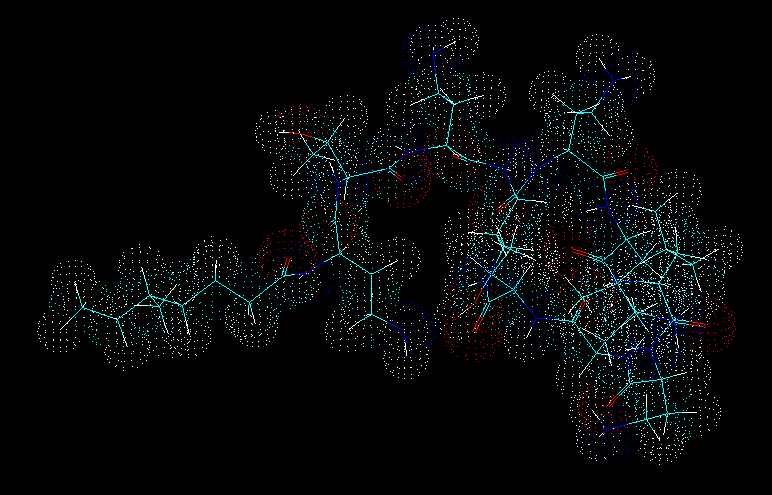 | 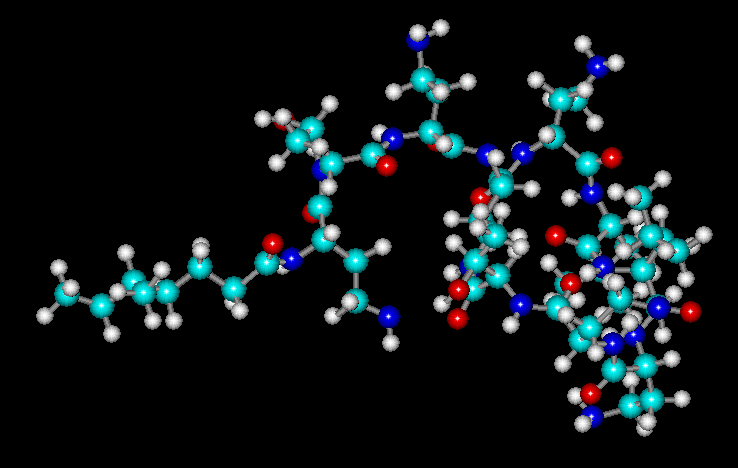 |
| **alexidine** | |
| 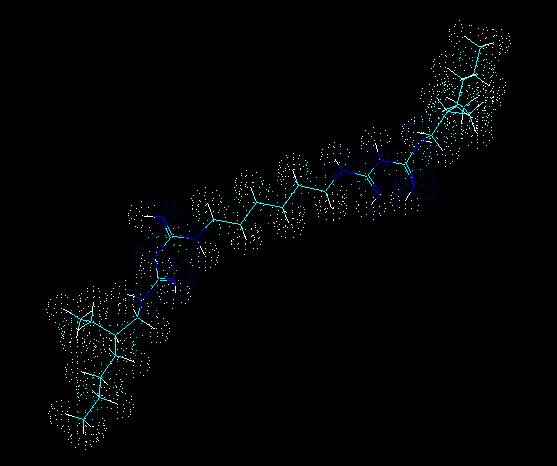 | 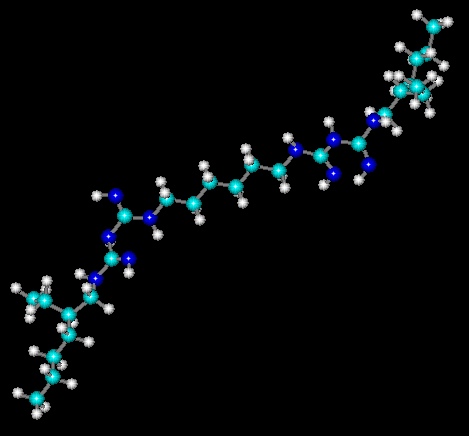 |

**Supplementary Figure 13.** **Rate of NPN uptake in *P.aeruginosa.* a.** Kinetics of NPN uptake in PAO1 in the absence or in the presence of NV716**.** **b.** Rate of NPN uptake over the early time point (0-4s) in 4 *P.aeruginosa* strains (2 reference strains [PAO1 and PAO509] and two colistin-resistant isolates [PA2938 and PA307]). All data are the mean ± SEM (triplicates from three independent experiments). Statistical analysis: 1-way ANOVA with Tukey post-hoc test: data series with different letters are different from one another (p<0.05).

The rate of NPN uptake was 70% higher in PAO509 than in PAO1, but 55-60% lower in colistin-resistant isolates PA2938 and PA307 than in PAO1.

**Supplementary Figure 14. Fluorescence of NV1532. a.** Fluorescence emission spectrum of NV1532 at an excitation wavelength of 470nm and **b.** calibration curve showing the quantitative relationship between the fluorescence signal and the concentration of NV1532 in PBS. The fluorescence signal of PBS is deduced from all values (background). All values are the means from three independent experiments.

**Supplementary method S1: synthesis and characterization of NV1532**

All solvents were purified according to reported procedures, and reagents were used as commercially available. Methanol, ethyl acetate, dichloromethane, and petroleum ether (35-60°C) were purchased from Merck and used without further purification. Column chromatography was performed on Macherey-Nagel silica gel (70-230 mesh). ^1^H NMR and ^13^C NMR spectra were recorded in CDCl_3_ or CD_3_OD on a Bruker AC 400 spectrometer working at 400 MHz and 100 MHz, respectively (the usual abbreviations are used: s: singlet, d: doublet, t: triplet, q: quadruplet, m: multiplet). All chemical shifts are given in ppm.

The synthesis was performed as outlined in **supplementary scheme 1.**

Supplementary scheme 1: synthetic scheme for NV1532.

**5-(4-(tert-butoxycarbonylamino)piperazine-1-carbothioamido)-2-(6-hydroxy-3-oxo-3H-xanthen-9-yl)benzoic acid (3)**: In a two-necked round flask were placed 110 mg of fluorescein isothiocyanate **1** (2.8 10^-4^ mol) and 57 mg tert-butyl piperazin-1-ylcarbamate (2.8 10^-4^ mol) in 5 mL of dimethylformamide. 5 mL of triethylamine were then added (5.6 10^-4^ mole) and the reaction mixture was stirred at room temperature for 12 h. Saturated sodium chloride solution and ethyl acetate was added to the reaction mixture and the aqueous layer was extracted twice with ethyl acetate. The combined organic layers were dried over anhydrous sodium sulfate and concentrated on rotary evaporator. The crude product was obtained as an orange solid used as it is in the next step. NMR ^1^H (400 MHz, CDCl_3_): δ (ppm) = 8.04 (m, 4H), 7.78-7.80 (m, 1H), 7.18-7.20 (m, 1H), 6.76-6.78 (m, 4H), 6.61-6.64 (m, 2H), 4.87 (s, 1H), 4.15-4.17 (m, 4H), 2.91-2.95 (m, 4H), 1.54 (s, 9H). NMR ^13^C (100 MHz, CDCl_3_): δ (ppm) = 183.15, 170.96, 161.33, 157.02, 153.97, 148.95, 143.73, 133.08, 130.14, 128.53, 125.02, 121.25, 113.65, 111.34, 103.44, 81.01, 55.62, 36.99, 28.67. MS (ESI^+^): m/z 591.1869 ([M+H]^+^).

**5-(4-aminopiperazine-1-carbothioamido)-2-(6-hydroxy-3-oxo-3H-xanthen-9-yl)benzoic acid (4)**: In a two-necked round flask were placed under argon 3 g of acetyl chloride in 10 mL of Methanol to generate a 4 N hydrochloric acid solution. The mixture was stirred for 4 hours at room temperature then 150 mg of crude derivative **3** were added. The reaction mixture was stirred at room temperature for 36 h then concentrated under vacuum. Ethyl acetate was added to precipitate the product as the hydrochloride salt which was neutralized with trimethylamine and carried forward without further purification (106 mg, 87% yield).

**Fluorescent rifampicin (5):** 155 mg of 3-Formyl rifamycin (2.1 10^-4^ mol), 106 mg of **4** (2.1 10^-4^ mol) and 45 mg of triethylamine (4.2 10^-4^ mol) were dissolved in 5 mL of methanol. The reaction mixture was stirred at room temperature for 36 h then concentrated under vacuum. The crude product was purified by flash chromatography on silica gel (dichloromethane: methanol 1:0 to 9:1) to yield the expected product **5** as an orange-brown solid (75 mg, 33%). The purity of the final product was >95% as determined by HPLC analysis conducted on an Agilent 1100 HPLC/MS system (C18 column, 5 μm, 3 x 50 mm, 0.45 mL/min, UV 254 nm, room temperature) with gradient elution (5-95% methanol in water over 10 min with all solvents containing 0.05% trifluoroacetic acid). NMR ^1^H (400 MHz, CD_3_OD): δ (ppm) = 9.98 (s, 1H), 9.28 (s, 1H), 8.05-8.27 (m, 2H), 7.42-7.64 (m, 3H), 5.86-6.59 (m, 9H), 5.59-5.82 (m, 2H), 4.56-4.78 (m, 2H), 3.19-4.15 (m, 18H), 1.74-2.48 (m, 17H), 0.778-1.05 (m, 12H). ^13^C NMR (125 MHz, CD_3_OD) δ 188.36, 182.79, 181.07, 177.45, 172.18, 171.98, 170.68, 168.38, 161.13, 155.45, 154.65, 154.47, 143.77, 143.11, 142.95, 140.98, 137.66, 137.42, 133.57, 132.76, 129.06, 128.60, 128.41, 128.13, 127.89, 126.29, 125.93, 119.40, 119.31, 118.62, 117.68, 117.05, 116.77, 115.31, 112.42, 112.08, 111.49, 109.87, 109.67, 104.02, 103.09, 100.94, 82.21, 77.16, 73.85, 73.30, 56.63, 48.60, 47.49, 42.79, 38.90, 37.23, 34.10, 21.91, 21.85, 20.74, 17.85, 10.88, 10.08, 9.49, 7.47. MS (ESI^+^): m/z 1198.3070 ([M+H]^+^).

**Supplementary references**

1. Sautrey, G. *et al.* New amphiphilic neamine derivatives active against resistant Pseudomonas aeruginosa and their interactions with lipopolysaccharides. *Antimicrob Agents Chemother* **58**, 4420-4430 (2014).

2. Jeannot, K. *et al.* Detection of Colistin Resistance in Pseudomonas aeruginosa Using the MALDIxin Test on the Routine MALDI Biotyper Sirius Mass Spectrometer. *Front Microbiol* **12**, 725383 (2021).
