# Supplementary material for: The membrane-active polyaminoisoprenyl compound NV716 re-sensitizes Pseudomonas aeruginosa to antibiotics and reduces bacterial virulence
Source: Commun Biol. 2022 Aug 25;5:871. doi: 10.1038/s42003-022-03836-5 (PMC9411590; doi:10.1038/s42003-022-03836-5)
Supplement: Supplementary file 2 — Description of Additional Supplementary Files [file 42003_2022_3836_MOESM2_ESM.docx]

**Description of Additional Supplementary Files**

**File name:** Supplementary Data 1
**Description:** MIC of antibiotics alone or combined with potentiators against P. aeruginosa reference strains and clinical isolates.

**File name:** Supplementary Data 2 **Description:** source data underlying Fig 1-4, 5a-e, 6d, 7-8, 9c-f.
