# Supplementary material for: The membrane-active polyaminoisoprenyl compound NV716 re-sensitizes Pseudomonas aeruginosa to antibiotics and reduces bacterial virulence
Source: Commun Biol. 2022 Aug 25;5:871. doi: 10.1038/s42003-022-03836-5 (PMC9411590; doi:10.1038/s42003-022-03836-5)
Supplement: Supplementary file 5 — Reporting Summary [file 42003_2022_3836_MOESM5_ESM.pdf]

nature portfolio

Corresponding author(s): VAN BAMBEKE Françoise

Last updated by author(s): 09/08/2022

Reporting Summary

Nature Portfolio wishes to improve the reproducibility of the work that we publish. This form provides structure for consistency and transparency in reporting. For further information on Nature Portfolio policies, see our Editorial Policies and the Editorial Policy Checklist.

Statistics

For all statistical analyses, confirm that the following items are present in the figure legend, table legend, main text, or Methods section.

n/a	Confirmed	
☐	☒	The exact sample size (n) for each experimental group/condition, given as a discrete number and unit of measurement
☐	☒	A statement on whether measurements were taken from distinct samples or whether the same sample was measured repeatedly
☐	☒	The statistical test(s) used AND whether they are one- or two-sided<br>*Only common tests should be described solely by name; describe more complex techniques in the Methods section.*
☒	☐	A description of all covariates tested
☒	☐	A description of any assumptions or corrections, such as tests of normality and adjustment for multiple comparisons
☐	☒	A full description of the statistical parameters including central tendency (e.g. means) or other basic estimates (e.g. regression coefficient) AND variation (e.g. standard deviation) or associated estimates of uncertainty (e.g. confidence intervals)
☐	☒	For null hypothesis testing, the test statistic (e.g. F, t, r) with confidence intervals, effect sizes, degrees of freedom and P value noted<br>*Give P values as exact values whenever suitable.*
☒	☐	For Bayesian analysis, information on the choice of priors and Markov chain Monte Carlo settings
☒	☐	For hierarchical and complex designs, identification of the appropriate level for tests and full reporting of outcomes
☒	☐	Estimates of effect sizes (e.g. Cohen's d, Pearson's r), indicating how they were calculated

Our web collection on statistics for biologists contains articles on many of the points above.

Software and code

Policy information about availability of computer code

Data collection	no code was used.
Data analysis	Statistical analyses and curve fitting were performed using GraphPad Prism software for Windows (version 9.0.0; GraphPad Software, Inc., San Diego, CA).<br>DNAseq: The obtained reads were quality controlled by “fastqc” with default settings. The tool suite “bbtools” was used for cleaning and merging reads, and for performing statistical analyses on coverage and insert sizes (BBMap – Bushnell B. – sourceforge.net/projects/bbmap/). The trimmed reads were de-novo assembled using “shovill” (https://github.com/tseemann/shovill) with the assembler “SPAdes” with the optional setting ‘only-assembler’. Single nucleotide polymorphisms (SNPs) were detected by “parsnp” with default settings. All references are given in the paper.<br>RNAseq: The obtained reads were quality controlled (option “--nextseq-trim=20”) and clipped using cutadapt. Reads were mapped to the reference genome PAO1 (NC_002516.2) using bowtie2. The resulting sam-files were converted to indexed binary format using SAMtools. Counts of the mapped reads were extracted with featureCounts using reads with a minimum mapping quality of 20. Differential gene expression analysis was performed with the R package edgeR. Normalization factors to scale the raw library sizes were calculated using the weighted trimmed mean of M-values (TMM) method. Dispersions were estimated and data were fitted using the edgeR functions estimateDisp and glmQLFit, respectively. All references are given in the paper.

For manuscripts utilizing custom algorithms or software that are central to the research but not yet described in published literature, software must be made available to editors and reviewers. We strongly encourage code deposition in a community repository (e.g. GitHub). See the Nature Portfolio guidelines for submitting code & software for further information.

Data

Policy information about availability of data

All manuscripts must include a data availability statement. This statement should provide the following information, where applicable:

- Accession codes, unique identifiers, or web links for publicly available datasets
- A description of any restrictions on data availability
- For clinical datasets or third party data, please ensure that the statement adheres to our policy

DNAseq: The raw sequencing files have been deposited in the NCBI SRA database under the accession number PRJNA785668.
RNAseq: The raw sequencing files have been deposited in the NCBI GEO database under the accession number GSE190194.
Other data: All other raw data will be available from the corresponding author upon request.

Field-specific reporting

Please select the one below that is the best fit for your research. If you are not sure, read the appropriate sections before making your selection.

☒ Life sciences ☐ Behavioural & social sciences ☐ Ecological, evolutionary & environmental sciences

For a reference copy of the document with all sections, see nature.com/documents/nr-reporting-summary-flat.pdf

Life sciences study design

All studies must disclose on these points even when the disclosure is negative.

Sample size	There was no statistical pre-determination of sample size. Sample sizes were defined based on prior experience with similar types of experiments. As indicated here below, each experiment was performed at least three times independently with at least three replicates (rarely two replicates).
Data exclusions	No data were excluded from the analyses.
Replication	M Most experiments were performed at least in triplicates at 2 to 4 independent occasions (see details in each figure legend) and data presented as the mean and SEM when applicable. Three samples were prepared for transcriptomic analyses and one sample for genomic or MS analyses. Confocal microscopy analyses were performed on three independent samples with data presented as the mean value; representative images from one sample are shown. Electron microscopy was performed on a single sample but with examination of different zones randomly selected.
Randomization	Not applicable for the data presented in this study . Experiments were carried out on cultures obtained from a single subculture within one experiment. All replicates within a single experiment could thus be considered as sharing the same characteristics.
Blinding	Investigators were not blinded, but RNAseq and DNAseq analyses as well as electron microscopy or confocal microscopy were performed by other researchers than the other experiments. These researchers were not blinded because they were not aware of the other data collected independently by the main author of the study. There was thus no risk of influencing their analysis of their own data.

Reporting for specific materials, systems and methods

We require information from authors about some types of materials, experimental systems and methods used in many studies. Here, indicate whether each material, system or method listed is relevant to your study. If you are not sure if a list item applies to your research, read the appropriate section before selecting a response.

Materials & experimental systems

n/a	Involved in the study	
☒	☐	Antibodies
☒	☐	Eukaryotic cell lines
☒	☐	Palaeontology and archaeology
☒	☐	Animals and other organisms
☒	☐	Human research participants
☒	☐	Clinical data
☒	☐	Dual use research of concern

Methods

n/a	Involved in the study	
☒	☐	ChIP-seq
☒	☐	Flow cytometry
☒	☐	MRI-based neuroimaging
